# Wheat Flour Quality Assessment by Fundamental Non-Linear Rheological Methods: A Critical Review

**DOI:** 10.3390/foods12183353

**Published:** 2023-09-07

**Authors:** Gamze Yazar

**Affiliations:** Department of Animal, Veterinary and Food Sciences, University of Idaho, Moscow, ID 83844, USA; gamzey@uidaho.edu

**Keywords:** wheat quality, wheat flour dough, physical dough testing, non-linear rheology

## Abstract

Wheat quality assessment involves physical, physicochemical, chemical, and sensory characterization of wheat kernels and the resulting wheat flour, dough, and bread. The physical tests conducted on wheat flour dough are mostly based on empirical methods. Empirical methods have been useful in industry and research to relate wheat flour quality to baking performance. However, these methods have the disadvantage of providing data in arbitrary units, which makes the fundamental interpretation of results difficult. Therefore, this review focuses on the use of fundamental rheological methods to determine wheat flour quality in terms of processing performance. During the transition from wheat flour to bread, wheat flour dough is mostly exposed to large deformations, and the quality of wheat flour determines its response to these large deformations and its baking quality. For this reason, this review only focuses on the application of fundamental rheological tests that are conducted in the non-linear viscoelastic region where wheat flour dough experiences large deformations.

## 1. Introduction

Wheat quality assessment is crucial for the milling and bakery industry for the production of baked products with standard quality. Numerous wheat varieties exist with various characteristics in terms of yield and resistance to insects and diseases, but also in terms of grain quality suitable for processing, such as milling and baking [1,2,3]. Moreover, the resulting variability in wheat flour quality makes it a challenge to bake over a longer period of time with constant recipes and constant process parameters [4]. To achieve standard quality in the final product, a series of physical, physicochemical, chemical, and sensory tests is conducted on wheat kernel, wheat flour, dough, and baked products. Among these wheat quality assessment methods, this review focuses on the physical dough testing methods, which are based on the rheological characterization of wheat flour doughs.

During breadmaking, a wide set of different physicochemical phenomena (i.e., gluten network formation, expansion of gas cells entrapped in the gluten–starch matrix, starch gelatinization, thermosetting of gluten proteins, etc.) occur, producing discernible changes in the rheological properties [5]. These changes occurring in the rheological response of wheat flour dough throughout the different stages of breadmaking have a major influence on baked product quality [6,7]. Therefore, the rheological characterization of wheat flour dough is essential to generate information regarding the quality of the raw material and the textural and sensory characteristics of the finished product [8]. The methods used for measuring the rheological properties of wheat flour dough have traditionally been divided into descriptive empirical techniques and fundamental measurements [9,10]. The most important empirical rheological methods include the Farinograph, Mixograph, Extensograph, Alveograph, Kieffer dough and gluten extensibility rig, and Rheofermentometer. These methods monitor the dough behavior during different processing operations, such as mixing, fermentation, and baking, while allowing the prediction of loaf volume as part of the baking quality [10,11,12]. Although empirical methods have demonstrated their usefulness in industry and research to relate the rheological behavior of dough to baking performance, they have the disadvantage of providing data in arbitrary units, which makes the fundamental interpretation of results difficult [9,13,14]. In these methods, shear, compression, and extension, as the basic types of deformation, occur simultaneously [12,15]. Moreover, the applied stress and strain states are uncontrolled, complex, and non-uniform. The geometry used is not well defined. Thus, it is impossible to define rheological properties through parameters in scientific units [9]. On the other hand, fundamental rheological testing methods are conducted using scientific instruments that are particularly designed so that their results can be expressed in terms such as stress, shear rate, strain, modulus, viscosity, etc. [13]. In contrast to empirical testing, only one type of deformation is applied during a fundamental rheological measurement. The advantages that come along with fundamental rheological methods include easy computation of the related physical properties, accurate comparison and interpretation of the data obtained, and the small number of samples required for testing [12].

A frequently used fundamental method for rheological testing of doughs is the Small Amplitude Oscillatory Shear (SAOS) test, which analyzes the linear viscoelastic response by observing the strain and frequency dependence of the elastic modulus (G′) and viscous modulus (G″) at small strains without disturbing the 3D structure of dough [16,17,18]. Small deformation tests are advantageous in understanding molecular interactions and microstructure [19]. However, during processing, wheat flour dough is mainly exposed to large deformations (i.e., mixing, fermentation, sheeting, oven-rise) at deformation rates ranging from low (i.e., from resting or fermentation) to high (i.e., mixing) [20,21,22]. Therefore, small deformation tests show little relationship with end-use performance as they are generally conducted under deformation conditions inappropriate for breadmaking [9,23]. Thus, fundamental rheological tests conducted under large deformations are required to characterize the viscoelastic properties of wheat flour dough during processing [6,21,24]. For example, wheat flour dough rheology needs to be characterized under conditions that are as close as possible to the real baking process, which implies studying changes occurring under increasing temperature and at large strains [7]. Alternatively, large deformations can be applied at frequencies or deformation rates ranging from low to high in order to approximate the deformations wheat flour dough experiences during mixing, sheeting, or proofing [21,25,26]. Moreover, certain dough samples may show similar linear rheological properties but can exhibit distinct nonlinear rheology, which is why probing nonlinear rheological properties can provide information that is not available from small deformation measurements [18,22,27]. Especially if the material contains high molecular weight (HMW) polymers [9], such as glutenin in wheat flour, measurements under large deformations often show very different rheological responses to those in small deformation. Studies have shown that fundamental large strain rheology could be used to differentiate different types of wheat flours, while small strain rheology was unable to differentiate between functionally very different flours [8]. Fundamental methods employed for exploring the mechanical properties of wheat flour dough under large deformations include capillary flow [28], lubricated squeezing flow [7,24,29,30], stress relaxation [7,31,32], stress growth [19], creep and creep recovery [24,33,34], and large amplitude oscillatory shear tests [6,21,26].

Several researchers have summarized the use of rheological methods to predict baked product quality. Dobraszczyk and Morgenstern [9] reviewed the applications of fundamental rheology in comparison with empirical rheology during the main steps of the breadmaking process to predict bread quality. The role of empirical rheology in flour quality control has been discussed by Hadnađev et al. [11]. Tietze et al. [12] discussed the possibility of linking the rheological properties of wheat flour dough characterized through empirical and fundamental methods, mainly focusing on the shear tests. Cappelli et al. [35] evaluated the changes occurring in the rheological response of wheat flour dough under different mixing configurations and suggested strategies to improve the resulting baked product quality. Della Valle et al. [15] provided an overview of the empirical and fundamental rheology methods used to reveal the microstructure and response of dough during mixing. On the other hand, the aim of this review is to show the possibility of employing fundamental rheological testing methods conducted under large deformations for the assessment of wheat flour quality.

## 2. Wheat Quality Assessment

The term quality for wheat is used in a very broad sense to define its overall potential to be successfully transformed into certain end products [36]. Therefore, wheat quality is generally assessed based on its suitability for the particular end-use [1]. Typically, about 95% of the wheat grown worldwide is hexaploid wheat [*Triticum aestivum* (common wheat) and *Triticum compactum* (club wheat)], while most of the remaining 5% is tetraploid wheat [*Triticum durum* (durum wheat)]. The latter is more adapted to the dry Mediterranean climate and is often called pasta wheat to reflect its major end-use, but it is also an important raw material for couscous and bulgur, particularly in North Africa and the Middle East [37,38,39]. In North American terminology, *T. aestivum* wheats are classified as soft and hard depending on their endosperm textures. Soft varieties are used for the production of cookies, crackers, cakes, and other baked products with a tender bite, while hard varieties are used for breadmaking. On the other hand, *T. compactum* has a very soft endosperm texture, and thus, it is used to produce very tender cookies [37,40].

**Figure 1:**
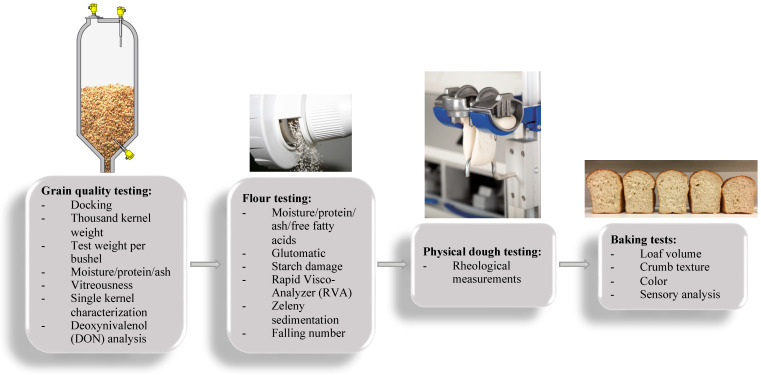
Wheat quality testing methods.

Apart from the genotype, the end-use quality of wheat is also related to environmental conditions (soil type and climate conditions), which may result in variability in wheat flour quality [36]. To be able to react pro-actively to the variations in wheat flour quality, bakery companies need reliable analytical methods that allow predicting the behavior of flour in production as well as the final bakery results [4]. From the bakers’ perspective, their first desire for the wheat flour they purchase is to have consistent performance for a consistent baked product quality [41]. As for the milling industry, wheat quality is linked to high extraction yield to maximize profit as wheat flour has a higher price than the milling by-products such as bran, germ, shorts, middlings, etc. Thus, undamaged wheat with little or no physical defects and functionality for a particular end use together constitute quality for the milling industry. The concept of quality for a farmer, on the other hand, is linked to a good germinability of the sown grain and a sound and undamaged harvest, free from sprout damage, disease, and insect pests that result in “defects”, and a moisture content assuring safe storage (generally, lower than 12%) [36,42]. Therefore, to bring a complete evaluation of wheat quality, a series of physical, physicochemical, chemical, and sensory analyses should be conducted on wheat kernels, wheat flour, wheat flour dough, and finally on baked products, as shown in Figure 1.

This review focuses on the application of fundamental non-linear rheological tests to determine wheat flour quality. For this reason, physical dough testing methods will be discussed in the next section.

## 3. Physical Dough Testing Methods

### 3.1. Rheology

Rheology is broadly defined as the field of science that studies the deformation and flow of materials with complex or non-Newtonian viscosity [43,44]. A rheological measurement is conducted on a given material by imposing a well-defined strain or strain rate and by measuring the resulting stress response or vice versa. The relationship between these physical events leads to different rheological properties that can be characterized by parameters such as modulus, viscosity, etc. [9,43]. Thus, rheology quantifies useful food descriptors such as creamy, mushy, slippery, rubbery, and astringent [44].

Basic concepts of fundamental rheology have been previously described in detail [43].

### 3.2. Classification of Material Behavior and Dough Rheology

Materials show two extreme responses depending on the relationship between strain and stress: ideal solid (elastic) and ideal fluid (viscous) behaviors [43]. An ideal solid (Hookean solid) deforms instantaneously when a load is applied and returns to its original configuration when the load is removed (complete recovery). On the other hand, an ideal fluid (Newtonian fluid) deforms at a constant rate when stress is applied, and it does not regain its original configuration as the load is removed [43,45].

Wheat flour dough is a viscoelastic system as it shows the characteristics of both elastic and viscous behavior [11]. When a piece of dough is placed on a flat surface in an environment with adequate relative humidity (%*RH*) to prevent its surface from drying, it flows. The degree of flow depends on the balance of viscous to elastic properties. On the other hand, when a piece of dough is stretched, and the force is released rapidly, it partially recovers its original shape due to its elastic recovery properties [40].

### 3.3. Importance of Rheology for Wheat Flour Quality

**Figure 2:**
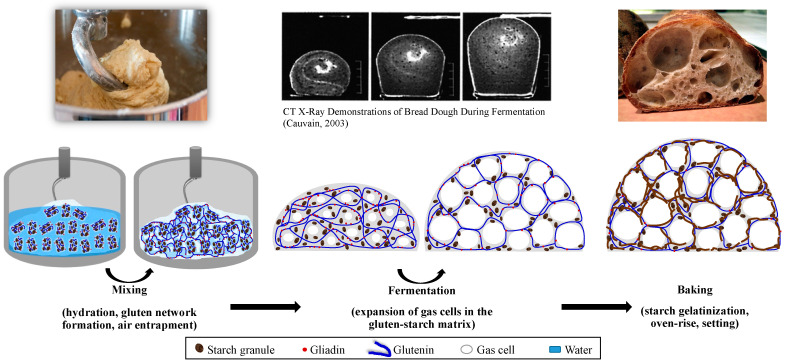
An illustration of the physical changes occurring in wheat flour dough at different stages of breadmaking. CT X-Ray dough fermentation images were reproduced with permission from Cauvain [57].

Wheat flour dough is the basis of many baked products such as bread, crackers, cakes, and cookies. Determining its rheological properties at different stages of processing is important in terms of predicting baked product quality [23]. In wheat flour quality testing, the use of physical dough testing methods is based on a “three-phase” system, the concept of which reflects the relevance of the individual physical qualities of dough at the three principal stages of the baking process: dough mixing, fermentation and handling, and oven rise during baking [35,46]. Figure 2 shows the transformation of wheat flour into a viscoelastic dough system and then into a solid-like baked product.

During mixing, the dough is exposed to shear and uniaxial extension deformations through the applied mechanical energy [8,47]. Under the exposure of large deformations, water is thoroughly distributed to hydrate the wheat flour particles, and thus, protein and starch are released to form the mobile phase and enable gluten network formation. As mixing proceeds, mechanical forces stretch the large molecules, particularly glutenin, and bring them to an extended configuration that aligns these molecules and promotes the formation of non-covalent bonds. The formation of intermolecular disulfide bonds results in polymerization and imparts elasticity to dough that improves its machinability and gas retention capacity [15,48,49]. As a result, the dough is developed into a 3D viscoelastic structure with gas-retaining properties (Figure 2), as gluten network formation is the primary stabilization mechanism for gas retention during leavening [22,50]. Moreover, air is entrapped in the gluten–starch matrix to form the nuclei for the gas cells that expand during fermentation [15,32,51].

During fermentation, carbon dioxide is produced by the yeast in the aqueous phase of the dough. As fermentation proceeds, the aqueous phase becomes saturated, and CO_2(g)_ starts to diffuse into the gas cells entrapped during mixing. These gas cells in the gluten–starch matrix expand progressively due to the increasing pressure of diffusing CO_2(g)_ [40,52]. Rheology of the gluten-starch matrix is important for the end-quality of leavened baked products as this determines extensibility and strength [50]. Expansion of the gas cells deforms the dough through biaxial extension at relatively lower deformation rates compared to those experienced during mixing [19,47,53], and this causes thinning of the dough film surrounding the gas cells [50]. Under these deformations, the extended gluten-starch matrix around the gas cells was suggested to be prevented from rupturing by a phenomenon called strain stiffening [54,55]. Strain stiffening is simply defined as the stress developed by the protein–starch matrix against the deformation resulting from the expanding gas cells. If the strain stiffening behavior of a dough system is above or below the optimum, a decrease is expected to occur in the loaf volume of the resulting baked product [22,52]. The viscoelastic nature (viscous to elastic ratio) of wheat flour dough determines the degree of strain stiffening behavior under large deformations and, thus, the degree of dough expansion and loaf volume.

Dough continues to behave as a viscoelastic material during most of the baking step [54]. As the temperature rises, increased yeast activity up to 55 °C and thermal expansion of water, CO_2(g)_, and ethanol contributes to oven-rise (Figure 2). As in fermentation, the gluten–starch matrix continues to show strain-stiffening behavior as it is stretched thin to cover the expanding gas cells, and the dough is exposed to biaxial extension during oven-rise. Therefore, the breadmaking performance of wheat flour is mostly determined by rheological methods based on biaxial extension [56]. In the later stages of baking, starch starts to gelatinize at around 65 °C. At temperatures above 88 °C, cell wall failures are observed due to progressive expansion, and finally, heat causes the gluten proteins to become highly cross-linked through the formation of disulfide bonds and the system sets [40]. The degree of oven spring has been regarded as an indicator of dough strength, meaning weak wheat flours with low gluten quality and quantity show little or no oven spring [57]. Two apparently equivalent doughs proofed to the same height may result in loaves with significantly different volumes, suggesting the heat-induced changes occurring in dough rheology during baking may define wheat flour quality [58].

As stated above, wheat flour dough is exposed to different types of deformations at each step of processing [24,47]. Depending on wheat flour quality, the response of wheat flour dough against these varying deformations changes. Moreover, the extent of these deformations should be optimized considering wheat flour quality to achieve improved baked product quality. For this reason, dough rheology constitutes an important part of wheat flour quality assessment.

Rheological methods based on different deformations to measure certain physical properties of wheat flour dough are discussed in the next section.

### 3.4. Classification of Rheological Methods

**Figure 3:**
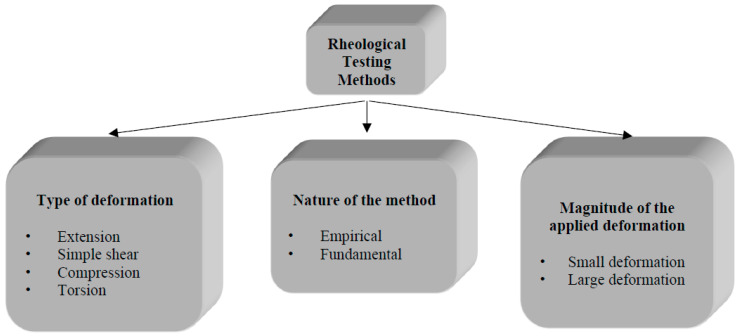
Classification of rheological testing methods.

Rheological tests attempt to measure the forces required to produce given controlled deformations. These test methods are commonly characterized according to the nature of the method, such as fundamental and empirical; the type of deformation, such as compression, extension, simple shear, and torsion; and the magnitude of the imposed deformation, such as small or large deformation (Figure 3) [9,43].

**Figure 4:**
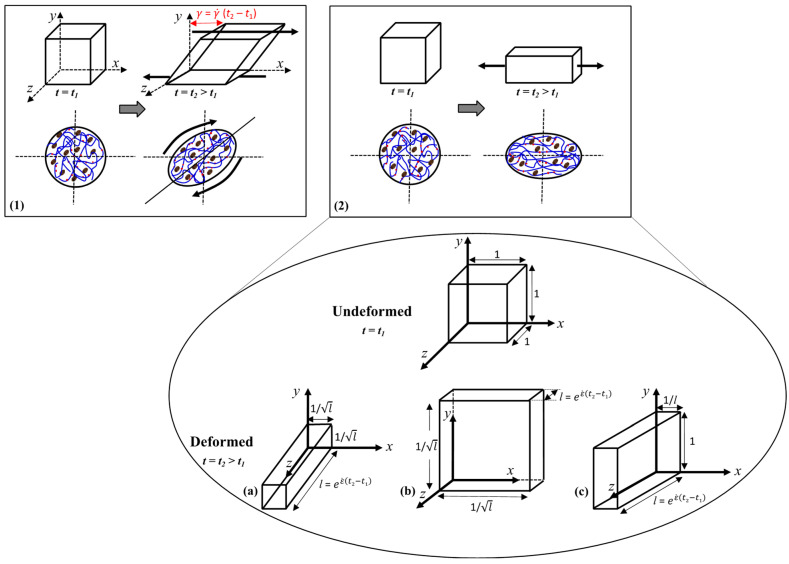
Types of deformations shown over the deformation of a unit cube of material and a dough piece with the macromolecules [starch granules (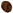), glutenin (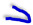) and gliadin (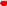) proteins] from time *t*_1_ to *t*_2_ (*t*_2_ > *t*_1_): (**1**) Shear, (**2**) Extension [(**a**) uniaxial, (**b**) biaxial, (**c**) planar]. The volume of material remains the same in all of these deformations. Reproduced with permission from Bird et al. [59] and Menjivar [20].

As stated above, dough processing mainly involves shear and extensional deformations [6]. In simple shear deformation, a material element is placed between two parallel plates where the bottom plate is stationary, and the upper plate is displaced in x-direction by Δ*x* by applying a force *F* tangentially to the surface (Figure 4(1)). On the other hand, pure extensional deformation does not involve shearing. There are three types of extensional deformation: uniaxial (Figure 4(2)(a)), biaxial (Figure 4(2)(b)), and planar (Figure 4(2)(c)). In uniaxial extension, the material is stretched in one direction, and this results in a corresponding size reduction in the other two dimensions. In biaxial stretching, a flat sheet of material is stretched in two directions with a corresponding decrease in the third direction. In planar extension, the material is stretched in one direction with a corresponding decrease in thickness while the height remains unchanged [43].

The main techniques used for measuring cereal properties have been traditionally divided into descriptive empirical techniques and fundamental measurements [9]. Empirical dough testing methods have been developed to monitor dough behavior during different processing operations such as mixing, kneading, molding, fermentation, and baking [11]. Recording mixers such as the Mixograph and Farinograph employ large deformations involving shear and extension to develop and subsequently demolish dough structures [53]. They measure the torque developed during dough mixing and produce a consistency curve that presents a peak indicating the optimum development of the dough [15,43]. A relatively newer dough testing device, Mixolab, measures dough behavior during mixing and heating and enables probing the contributions of wheat flour constituents to dough rheology in a single test. Thus, it performs continuous measurements throughout a simulated baking process [11].

Uniaxial (stretching) and biaxial (inflation) deformations are applied to dough in many empirical dough testing methods [5,6]. The bubble inflation method is the most popular in the dough industry as it simulates the biaxial expansion of gas cells during proof and oven rise. In this technique, a thin circular material sheet is clamped around its perimeter and inflated using pressurized air. Considering the importance of the bubble inflation method in the baking industry, a commercial test rig known as an Alveograph was developed [43]. The Alveograph measures the pressure required to rupture a dough sheet, whereas the area under the recorded pressure curve is related to the strength of wheat flour dough [15] and, thus, to the baking quality of wheat flour [53]. Kieffer dough and gluten extensibility rig and Extensograph methods are both based on measuring the uniaxial rheological behavior of dough samples. In the Extensograph, the dough sample is extended with a hook downwards, while the sample is extended upwards in the Kieffer dough and gluten extensibility rig attached to a texture analyzer [43]. The Extensograph measures the force required to stretch a dough cylinder down its middle to determine its resistance [15]. Proofing properties of dough (gas production and retention) can be monitored by a Rheofermentometer [11]. The Rheofermentometer monitors the gas production and retention capacities of dough and predicts its ability to expand during fermentation [15]. Finally, the empirical methods used to monitor starch gelatinization under shear include an Amylograph and Rapid-Visco Analyzer (RVA). Pasting, gelatinization, and setback viscosity properties during cooking and cooling can be obtained using these methods [5,11].

In order to fully understand the rheological response of dough and its relation to microstructure or to develop a constitutive theory that predicts the rheological response of dough, fundamental rheological testing should be conducted under several conditions, including different types of deformations, a broad range of deformation rates, and testing temperatures [23,25]. Empirical dough testing methods involve large deformations as they are designed to measure the changes occurring in the rheological properties of dough under deformations similar to those experienced during certain steps of dough processing. However, fundamental rheological methods can be conducted under both small and large deformations (Figure 3).

Linear viscoelastic properties of dough are mostly determined by the fundamental Small Amplitude Oscillatory Shear (SAOS) tests conducted under small deformations [16,17,60]. SAOS measurements have the limitation of not being appropriate in practical processing situations due to the rates at which the test can be used [43]. For instance, wheat flour dough has been shown to display linear viscoelastic behavior below the strain amplitudes of around 0.2%, which may slightly change depending on wheat flour quality [16]. However, the strain amplitudes experienced by the dough during breadmaking can range from 100% during sheeting to 1000% during fermentation and oven rise and up to 500,000% during mixing. Therefore, characterization of the non-linear rheological properties of dough systems may offer a more detailed understanding of their rheological responses under real processing conditions [16,17,20]. Fundamental non-linear rheological testing methods offer quantitative measures of the deformation dough experiences under controlled conditions [9]. Apart from the advantages fundamental large deformation tests offer in terms of characterizing the processing quality of wheat flours in comparison to empirical and small deformation tests, they have certain drawbacks, as listed in Table 1. The instrumentation for fundamental rheological testing was reported to be expensive [9]. However, a small amount of sample requirement [33] and data acquisition under controlled deformations [12], leading to prompt and accurate interventions in industrial productions, might compensate for the instrumentation expenses in the long run. Dobraszczyk and Morgenstern [9] suggested difficulties in maintaining the instruments for fundamental rheological testing in industrial environments. However, a lot has changed in the last 20 years, and smart technologies such as computer vision and artificial intelligence have started to pervade the food industry [61,62]. Thus, today, it should be easier to use scientific rheometers for industrial applications such as quality control or product development. On the other hand, phenomena such as wall slip and inertia might occur during fundamental non-linear rheological testing due to the large deformations applied, leading to inaccuracy in the obtained data. These drawbacks could be prevented or eliminated, as suggested in Table 1. Therefore, this review focuses on the possibility of using fundamental non-linear rheological methods to bring a more solid characterization of wheat flour dough rheology under processing conditions.

Fundamental non-linear rheological methods include lubricated squeezing flow, non-linear creep and creep recovery, and Large Amplitude Oscillatory Shear (LAOS) tests. The lubricated squeezing flow test applies biaxial extension by compressing the sample between parallel plates under lubricated conditions and thus provides data to predict the baking performance of wheat flour during proofing and oven rise [63]. Creep and creep recovery tests indicate the presence of non-linear behavior; however, they cannot provide a quantitative measure of the type and extent of the non-linear behavior [17,64]. A relatively recent technique, LAOS tests have been recently used to bring a quantitative measure of the non-linear viscoelastic properties of materials through the meaningful LAOS parameters [*G*′*_M_*, *G*′*_L_*, *η*′*_M_*, *η*′*_L_*], dimensionless LAOS parameters [*S*, *T*], Lissajous–Bowditch curves, and *e* and *v* Chebyshev coefficients that cannot be obtained with SAOS testing. LAOS tests involve the systematic increase of the amplitude of the applied strain or stress at fixed frequencies and measuring the stress or strain response. These tests apply shear and determine non-linear material response beyond the linear viscoelastic region [16,17,65]. A detailed overview of LAOS theory [66] and its applications on semisolid foods, including wheat flour doughs [16], have been previously provided.

**Table 1:** Advantages and drawbacks of fundamental non-linear rheological testing methods.

Advantages	Drawbacks
Provides quantitative measures through parameters such as stress, strain, strain rate, modulus, or viscosity. ^a,b^ Only one type of controlled and uniform deformation is applied. ^c^ Easy computation of related physical properties. ^c^ Accurate interpretation and comparison of the data. ^a^ Well-defined geometry. ^a^ Small quantities of sample requirement. ^c,f^ Ability to approach processing deformations under fully controlled test settings. ^e^	Expensive instrumentation. ^a^ Difficult to maintain in industrial environments because the instruments used are not as robust as those used in empirical testing. ^a^ Requires high levels of technical skills. ^a^ Wall slip may occur during testing, and thus, the data obtained can be inaccurate. ^b^ However, a slip can be prevented. ^d,e^ Inertia can be observed in oscillatory shear tests, but this can be eliminated by adjusting the test parameters, data corrections ^e^, or by using a small amount of sample. ^c^

^a^ Dobraszczyk and Morgenstern [9], ^b^ Campanella and Peleg [13], ^c^ Tietze et al. [12], ^d^ Yoshimura and Prud’homme [67], ^e^ Yazar et al. [16], ^f^ Wang and Sun [33].

## 4. Fundamental Non-Linear Rheological Methods for Wheat Flour Quality Assessment

### 4.1. Gluten Quality and Quantity

In wheat flour dough, rheological properties are associated with the quality and quantity of gluten proteins. Protein content, an important quality characteristic of wheat, affects the ability of wheat to produce bread and other baked products, and it is strongly affected by the environment and agricultural handling practices [14]. Within a cultivar, breadmaking quality increases linearly with increasing protein content, but for a given protein content, it is largely a function of the qualitative nature of gluten proteins [68]. Therefore, gluten quality and quantity characterization constitute a major part of wheat flour quality assessment in terms of baking performance.

Uthayakumaran et al. [8] studied the uniaxial extensional properties of wheat flour dough by extending the sample exponentially at a constant strain rate of 0.01 s^−1^ and calculated elongational viscosity using the following equation: *η_E_* = *σ/*$\dot{\varepsilon }$, where *σ* is stress and $\dot{\varepsilon }$ is the strain rate in the sample. At large strains that corresponded to elongating the dough around 270% of its original length, elongational viscosity increased rapidly, which was indicative of strain stiffening. Wheat flour doughs from wheat cultivars with different protein content (6–20%) showed higher elongational rupture viscosity and strain values as protein content increased. This was attributed to the increased strain stiffening behavior of wheat flour doughs as the protein content and glutenin ratio of wheat flour increased and was regarded as an indicator of improved breadmaking [8]. It has been suggested that wheat flour dough should show a certain level of strain stiffening for improved breadmaking quality [54]. However, a constant increase in the strain stiffening behavior with the increase in applied deformation was found to be detrimental to loaf volume [52]. These findings suggested improved breadmaking performance with increasing protein content in wheat flour. However, very high protein levels may limit the increase in loaf volume due to high strain stiffening behavior. Uthayakumaran et al. [8] also found an increase in the elongational rupture viscosity and strain stiffening behavior of wheat flour doughs but a decrease in the rupture strain as the glutenin-to-gliadin ratio increased from 0.7 to 1.4. This result showed that wheat flour dough could be extended further before rupturing when gliadin dominated the system, confirming the contribution of gliadins to flow and extensibility and the contribution of glutenins to the strength and elasticity of the dough. Fevzioğlu et al. [69] supported this finding and reported an enhancement in the elasticity of gliadin composites with the addition of high molecular weight (HMW) glutenins. They conducted lubricated squeezing flow tests on gliadin composites with added HMW glutenins.

The results obtained by the fundamental extension test [8] were compared to the results obtained from the Extensograph test [70]. The extensibility measured by Uthayakumaran et al. [70] and the elongation rupture viscosity measured by Uthayakumaran et al. [8] were highly correlated when both protein content (*r* = 0.924) and glutenin-to-gliadin content (*r* = 0.903) varied. Similarly, Edwards et al. [71] also reported correlations between the parameters obtained from large deformation creep recovery tests conducted on flours of durum wheat cultivars with gluten strengths ranging from high to low and those obtained from the empirical dough testing methods Alveograph and Micro-Mixograph conducted on the same wheat flour samples. They found the large deformation creep maximum strain (11.93–16.06%) to be negatively correlating with the Micro-mixograph mixing time (MT: 2.51–3.03 min, *r* = −0.97) and mixing stability (MS: 13.6–17.5, *r* = −0.83) as well as negatively correlating with the Alveograph peak height to curve length ratio (*P/L*: 0.65–1.03, *r* = −0.77) and positively correlating with the work of deformation until rupture (*W*: 87.3 × 10^−4^–129.8 × 10^−4^ J, *r* = 0.90). These correlations suggested that the use of non-linear creep recovery tests provided successful discrimination of durum wheat cultivars of varying gluten strength while requiring fewer samples than traditional physical dough testing techniques. Recently, non-linear creep recovery tests were employed to determine the 3D-printability of wheat flour dough systems. The data obtained were fitted to Burger’s model, and little or no (elastic) recovery after applied stress in wheat flour cookie doughs was found to be unsuitable for 3D printing [72]. 3D printing offers numerous opportunities for innovative food design and new product formulations, and due to their unique rheological properties, cereal flours have been found to be suitable for 3D printing, especially for extrusion-based printers [73]. Therefore, 3D printing coupled with non-linear creep recovery tests could be used to optimize wheat flour blends or dough formulas for end products with improved quality considering the different gluten quality and quantity needs of end products (i.e., cookie, bread, pasta, etc.) in wheat flour.

**Figure 5:**
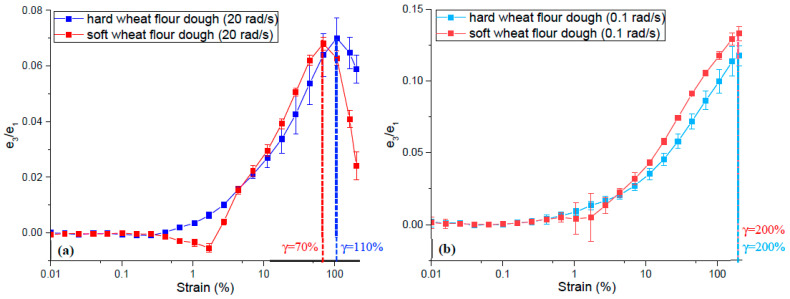
The ratio of the third-order elastic Chebyshev coefficient to the first-order (*e*_3_/*e*_1_) for hard and soft wheat flour doughs at high [20 rad/s (**a**)] and low [0.1 rad/s (**b**)] frequencies within the strain range of 0.01–200%.

A series of studies were conducted on wheat flour doughs made from hard red winter wheat [21] and soft red winter wheat [26] to determine their non-linear viscoelastic properties using the LAOS tests with an emphasis on the impact of gluten quantity in wheat flour on the dough’s rheological response to large deformations. The wet gluten contents of hard red winter wheat flour and soft red winter wheat flour were 29.8% and 23.9%, respectively. Both dough samples were obtained after 20 min of Farinograph mixing and then exposed to LAOS testing under strain amplitudes ranging from 0.01% to 200%. At the highest deformation frequency (20 rad/s) used in these studies, both hard and soft wheat flour doughs showed strain stiffening behavior in the non-linear region as evidenced by the positive ratio of the third-order elastic Chebyshev coefficients to the first order (*e*_3_/*e*_1_ > 0) (Figure 5a). The third-order Chebyshev coefficients provide physical insight into the deviation from non-linearity [66]. The sign of the third-order elastic Chebyshev coefficient indicates if the material shows strain stiffening (*e*_3_ > 0) or strain softening (*e*_3_ < 0). Moreover, the magnitude of *e*_3_/*e*_1_ provides a quantitative measure of the degree of non-linearity [74]. As the amplitude of strain increased, a decrease was observed in the magnitude of the strain stiffening behaviors. As shown in Figure 5a, this decrease was observed at a strain amplitude of 70% for soft wheat flour dough [26]; while it occurred at around 110% for hard wheat flour dough [21], suggesting an improved resilience for hard wheat flour dough against the applied deformations when compared to soft wheat flour dough with a lower gluten content. On the other hand, when the frequency of deformation was low (0.1 rad/s), no decrease was observed in the strain stiffening behaviors of hard and soft wheat flour doughs up to the strain amplitude of 200% (Figure 5b). This finding highlighted the impact of the frequency of deformation on the strain-stiffening behavior of wheat flour dough [21,26]. Due to its viscoelastic nature, wheat flour dough is known to partially recover after being stretched rapidly, and the force is immediately released [40]. In the LAOS tests conducted on hard and soft wheat flour doughs at high frequency (20 rad/s), the decay in the strain stiffening behavior observed at a certain strain amplitude, which was lower for soft wheat flour dough (Figure 5a), was attributed to the lack of time for the dough sample to recover under high-frequency deformations. Thus, the gluten network might undergo bond ruptures as the amplitude of strain kept increasing [21,26]. These findings obtained through the LAOS tests indicating the effect of wet gluten content in wheat flour on the strain stiffening behavior of the resulting dough cannot be obtained quantitatively using the empirical dough testing methods.

LAOS test was also used by Erturk et al. [18] to unravel the impact of protein content in wheat flours on the viscoelastic properties of the resulting doughs under large deformations. In this study, a different approach was used, and harmonical intensities (*I*) were evaluated to determine the MAOS (Medium Amplitude Oscillatory Shear) region and the onset of the LAOS region [18]. MAOS region can be defined as a transition region between SAOS and LAOS regions [75]. The analysis of the nonlinear region with the Fourier transform is complicated as it requires the inclusion of a greater number of harmonic intensity contributions of the Fourier Spectrum [76]. Therefore, analysis of the MAOS region has become a particular interest for the study of non-linear rheological behavior of materials [75]. The ratio of the third harmonic intensity to the first harmonic intensity (*I*_3_*/I*_1_) has been used to determine the boundaries of the MAOS region and, thus, the intrinsic non-linear properties of materials in this transition region [77,78]. The comparison of the second harmonic intensity to the third harmonics (*I*_2_*/I*_3_) has been shown as the confidence metric for too noisy MAOS data, while the ratio of the fifth harmonic intensity to the third harmonic intensity (*I*_5_*/I*_3_) has been suggested as the confidence metric for too non-linear MAOS data [79]. Using these metrics, Erturk et al. [18] provided an evaluation of the *I* values obtained from the LAOS tests (*ω*: 10 rad/s) conducted on soft wheat, hard wheat, and durum wheat flours. The lower strain limits (*I*_2_*/I*_3_ < 0.1) and the upper strain limits (*I*_5_*/I*_3_ > 0.1) of the MAOS region were 0.1–0.4% for soft wheat flour dough, 0.16–1.3% for hard wheat flour dough, and 0.27–2.2% for durum wheat flour dough. These values indicated that the MAOS region became longer as the protein content in wheat flour increased, suggesting a higher toleration against the increasing strain amplitudes for wheat flour doughs in the presence of higher protein content.

Using fundamental non-linear rheological testing methods, it is also possible to conduct measurements on gluten to directly measure its strength under large deformations. Uthayakumaran et al. [80] studied the non-linear viscoelastic properties of gluten through the elongation (uniaxial extension) tests and compared the results to those of wheat flour dough and reconstituted dough. Gluten doughs (55% water, *v*/*w*) showed higher elongational viscosity than wheat flour dough (39% water, *v*/*w*) mixed at optimum water content. As gluten was blended with increasing percentages of starch to obtain reconstituted dough, a gradual decrease was observed in elongational viscosity, indicating a decrease in the strain-stiffening behavior of dough in the presence of starch. When the gluten content dropped below 40% in reconstituted doughs, strain stiffening behavior started to disappear, which pointed out the impact of gluten and minor constituents such as lipids and water-soluble proteins on the elongational properties of dough [80]. Wheat flour contains 1.4–2.0% endogenous lipids, which play an important role in breadmaking since they contribute to the formation and development of bread loaf volume by positioning themselves in the gas cell interface to prevent the coalescence of bubbles [81]. The interaction between endogenous lipids and gluten has been reported to contribute to the strength of the gluten networks by promoting the interaction of hydrophobic amino acids by serving as binding agents and bridges. Since gluten network formation is the primary stabilization mechanism for gas retention during leavening, these networks’ strength and physical characteristics determine how much air can be incorporated into the dough [50,82]. To unravel the impact of endogenous wheat flour lipids on non-linear viscoelastic properties of the gluten network, Yazar et al. [22] conducted LAOS tests [*γ*: 0.01–200%, *ω*: 1, 10, and 20 rad/s] on gluten in the presence and absence of endogenous lipids. For this purpose, lipids were removed from vital wheat gluten using 95% ethanol. Both gluten and lipid-removed gluten were mixed in the Farinograph for 25 min with the same amount of added water (118.8% on a dry gluten basis). LAOS sweeps did not show a significant difference between the viscoelastic properties of gluten with and without lipids in the linear region at low frequency. The differences in the viscoelastic properties of gluten in the presence and absence of endogenous lipids became more evident under large deformations beyond the linear viscoelastic region of wheat gluten, where the molecular network was stretched, and networking weaknesses were magnified. Higher degrees of elasticity and strain stiffening were found for wheat gluten in the absence of lipids under large deformations. This was attributed to the stabilizing effect of lipids on the viscoelastic nature of the gluten network [22]. In the absence of lipids, the gluten network in a wheat flour dough system was reported to be more developed with a higher degree of crosslinking [22,83,84]. These findings showed the possibility of capturing the impact of minor components in wheat flour on dough viscoelasticity through the use of non-linear rheological testing methods, which cannot be determined by small amplitude oscillatory shear tests. The Farinograph tests conducted as an empirical dough testing method on gluten with and without endogenous lipids by Yazar et al. [22] revealed a higher affinity to water for gluten in the absence of lipids, as evidenced by the gradually increasing consistency during Farinograph mixing. The interactions of lipids with the side chains of gluten proteins were suggested to be the reason for the controlled water access to gluten proteins during mixing [22,84,85]. The findings through the empirical Farinograph tests provided information regarding the impact of endogenous wheat lipids on the consistency of gluten under undefined mixing deformations in the arbitrary unit of Brabender Units (BU). On the other hand, LAOS tests conducted within a defined strain range showed through the fundamental units of LAOS parameters how endogenous wheat lipids affected the viscoelastic response of gluten under large deformations at frequencies ranging from low to high that resemble the processing conditions [22]. Viscoelastic properties of a dough system during physical dough testing depend on how quickly the test is performed (depends on the applied shear rate or frequency). Furthermore, it is important to conduct dough rheology testing at strain amplitudes with a certain range of frequencies, which is possible with the LAOS technique. During processing, the dough is exposed to high-frequency deformation in the mixing or sheeting steps [9], while the frequency or rate of deformation is much smaller during proofing and oven rise [20,24].

LAOS deformation was also used by Ng et al. [86] to evaluate the mechanical properties of hydrated gluten (63% water on a total weight basis, *v*/*w*) through the Lissajous–Bowditch curves obtained from controlled oscillatory forcing under various deformation amplitudes (0.02–6) and frequencies (0.1–10 rad/s). In the non-linear region, Lissajous curves indicated a gradual softening of the network, as evidenced by the rotation of the major axis of the stress loop. The softening was suggested to reflect the reduction in network connectivity as the polymer chains are increasingly stretched under large shear strain. A distinct “stiffening,” indicated by the upturn of the shear stress, was observed at large strains. In order to study the recovery properties of the gluten network, Ng et al. [86] treated gluten with urea and subsequently washed away the urea with water. Apart from this chemical treatment, they conducted a mechanical treatment by the sudden increase of the applied strain followed by the subsequent decrease (*γ*_0_: 0.1 to *γ*_1_: 3 at time *t*_1_, and then back to *γ*_0_: 0.1). Both the chemical and mechanical treatments did not affect covalent interactions within the filaments, thus allowing a complete recovery of the network structure when the disruptions are removed. Under large deformations, the hydrogen bonds at the network junctions were suggested to be reversibly broken, and thus, upon the reduction of the imposed strain (at *t* = 169.6 s), hydrated gluten was able to reform its network structure due to the reversible nature of hydrogen bonds [86]. These findings were in line with the loop-train model proposed to describe the gluten network by Belton [87], where both the loop and train zones comprised hydrogen bonds. Although hydrogen bonds are much weaker than covalent bonds, their large number and ability to interchange under stress render them the main determinants of the mechanical properties of the gluten network [88]. Thus, concurring with the chemistry of the gluten network, Ng et al. [86] provided an in-depth characterization of the rheological responses of the gluten network under large deformations using the LAOS technique.

Uthayakumaran et al. [80] studied the stress relaxation responses of defatted gluten doughs (55% water, *w*/*v*) in the non-linear region (*γ*: 0.1%). They did not report a significant difference in the relaxation moduli, *G(t)*, for gluten doughs obtained from strong flour (13.9% protein) and baker’s flour (12% protein) during the relaxation time of 10^3^ s. On the other hand, Li et al. [31] also conducted stress relaxation tests on defatted gluten (66.67% water, *w*/*v*) in the non-linear region (*γ*: 5%) and reported a higher relaxation modulus for gluten from strong flour (10.6% protein) compared to gluten from weak flour (7.5% protein) over the whole relaxation time up to 10^3^ s. This was attributed to the stronger network structure, which could be due to entanglements and physical cross-links in the gluten from strong flour [31]. The different results provided by Uthayakumaran et al. [80] and Li et al. [31] can be due to the application of different strain amplitudes. It can result from the differences in the gluten samples studied. Uthayakumaran et al. [80] extracted gluten from wheat flour with high and low protein content. However, the wheat flour with lower protein content had a higher glutenin-to-gliadin ratio and required a longer time for optimum mixing. Thus, the gluten characteristics of the low-protein wheat flour they used might have overshadowed the stress relaxation data reported by Uthayakumaran et al. [80]. The breadmaking performance of wheat flour mainly depends on gluten quality and quantity, which may differ from one wheat variety to another. Moreover, the differences in gluten quality have been related to the glutenin to gliadin ratio, which determines the ratio of elastic to viscous properties in dough, and to glutenin quality, which improves dough cohesiveness and elasticity due to its high molecular weight, while contributing to expansion of the gas cells by preventing them from rupturing at the early stages of proofing [51,89]. Molecular weight has been shown to significantly impact relaxation time; the smaller the molecular weight, the shorter the relaxation time. A narrower molecular weight distribution results in a much sharper drop in the relaxation modulus [43]. Therefore, stress relaxation tests can be useful in distinguishing the baking performance of wheat flour doughs based on gluten quality [80].

Studies that relate wheat flour quality to the viscoelastic properties of gluten network have mostly focused on the impact of wheat flour components on the viscoelastic response of gluten-water and gluten-starch-water dough, while others have explored the individual effects of gluten proteins, gliadins, and glutenins [24]. Li et al. [31] studied the viscoelastic properties of hydrated gliadin (0.6 mL water/g) and glutenin (1 mL water/g) using stress relaxation tests conducted in the non-linear region (*γ*: 5%). Regardless of the origin of wheat flours being strong or weak, glutenin had higher stress relaxation modulus [*G(t)*] values with a longer relaxation time when compared to gliadin, indicating the higher molecular weight distribution in wheat glutenin. Yazar et al. [90] supported this finding through the Lissajous curves obtained as a result of the LAOS tests (*γ*: 0.1–200%; *ω*: 1 rad/s, 10 rad/s, 20 rad/s). Both elastic and viscous Lissajous curves for glutenin did not show a pronounced change as seen for gliadin against the increasing strain and frequencies, pointing out the flexible fluid structure of gliadin and the stiff character of glutenin. Glutenin showed a constant increase in the intra-cycle strain stiffening behavior in the non-linear region [27,90], while the degree of strain stiffening behavior for gliadin started to decrease beyond the strain amplitude of 110% [90] as evidenced by the Chebyshev coefficients. These findings on wheat gluten fractions helped us to understand the impact of the molecular structure of these protein fractions on their individual flow behaviors under large deformations. The intermolecular disulfide bonds in glutenin resulted in a strong network, and the intramolecular disulfide bonds coupled with secondary bonding interactions (hydrophobic, hydrophilic, and ionic) in gliadin resulted in a much weaker network structure against the increasing strain amplitudes [90].

Khatkar et al. [89] studied the linear viscoelastic properties of wheat glutenin through frequency sweeps [*τ_0_*: 318.3 Pa; *ω*: 0.1–10 Hz (=0.6–62 rad/s)] and reported *tanδ* values ranging from 0.12 to 0.5 for wheat glutenin extracted from strong wheat flour, where the protein content recovered in the gluten was 74.1%. On the other hand, Yazar et al. [27] evaluated the viscoelastic properties of wheat glutenin extracted from commercial vital wheat gluten with a protein content of 75.8% under large deformations using the LAOS tests (*γ*: 0.1–200%; *ω*: 1 rad/s, 10 rad/s, 20 rad/s) and reported *tanδ* values ranging from 0.19 to 1.07. Both wheat glutenin samples studied by Khatkar et al. [89] and Yazar et al. [27] were from wheat gluten with similar protein contents, but a higher degree of change in the *tanδ* values was found with the fundamental rheological test conducted in the non-linear region. These findings highlight the capability of non-linear rheological tests to capture changes in the viscoelasticity of wheat gluten fractions to a broader extent when compared to small amplitude oscillatory shear tests. However, coupling LAOS sweeps with small deformation frequency sweeps, as suggested by Le et al. [91], might provide deeper insight into the changes occurring in the gluten network microstructure after exposure to different magnitudes of deformation. In this method, LAOS sweeps were conducted up to a certain strain amplitude. The tests were stopped at select strain amplitudes, and after each LAOS sweep, a frequency sweep was conducted immediately in the linear viscoelastic region [91]. The coupled amplitude-frequency sweep method has not been conducted on wheat flour dough systems yet.

Non-linear rheological properties of gluten can also be determined through the empirical testing method, Kieffer dough, and gluten extensibility rig, that apply uniaxial extension and provides the parameters of maximum force (resistance to extension) and distance to break (extensibility) [43]. However, apart from providing an in-depth characterization through the application of other types of deformations in a controlled manner, fundamental non-linear rheological methods also enable the characterization of the viscoelastic properties of gluten fractions under large deformations, which cannot be obtained through the empirical dough testing methods.

### 4.2. Mixing Behavior

Most of the studies on doughs have focused on the relationships between mixing, rheology, and baking performance due to the rheological changes occurring in the gluten viscoelastic network during mixing and the importance of these changes on product quality [9,15]. Despite large changes in the dough rheology occurring during mixing, there is little information in the literature regarding the characterization of these changes at the different stages of the mixing process. Changes in the linear viscoelastic properties of dough with mixing time were found to be small [92]. Therefore, this section focuses on the use of fundamental rheological methods conducted on wheat flour doughs in the non-linear viscoelastic region to evaluate the baking performance of wheat flour through the changes occurring in dough rheology during mixing.

Characterization and accurate comparison of the dough’s rheological properties are complex as there is huge variability in wheat quality due to environmental changes or sourcing diversification [15], and different mixing processes involve different types of deformations [1,49]. As mentioned in Section 3.3, different mechanical actions, such as compression, shear, and extension, occur simultaneously and contribute to dough development [12]. Especially when high-speed mixers are used, the flow becomes very complex, and a combination of shear and extensional flow exists [92]. It has been demonstrated that fluid flow through an orifice can be represented as a combination of shear and extensional flow and, therefore, could more closely resemble the flow conditions in a dough mixer [92,93]. Therefore, Zheng et al. [92] studied the rheological changes occurring in wheat flour dough during mixing by conducting fundamental rheological methods that apply shear and extensional deformations on doughs prepared with two flours of different strength at various levels of mixing energy. They conducted extrusion tests (sample dimensions: 120 mm × 20 mm, extrusion diameter: 4 mm, extrusion rate: 60 mm/min) that combined shear and extension flows. Extrusion forces obtained for both soft and strong wheat flour doughs showed an increase up to 20 W.h/kg followed by a sharp decrease, indicating the disruptive impact of high mixing work input on the gluten–starch network. Even though relatively higher extrusion forces were obtained for strong wheat flour dough, the extrusion test was found to be inadequate in probing the variation among flours. Zheng et al. [92] also conducted shear tests under small (*γ*: 1%; *ω*: 0.01–10 Hz) and large deformations ($\dot{\gamma }$: 0.01–0.5 s^−1^) along with planar extensional tests (displacement: 80-–120 mm, speed: 60 mm/min, *ε*: 0–1%). It was not possible to obtain reliable results from the extension tests for soft wheat flour dough due to pronounced sagging. Strong wheat flour dough showed increasing extensional viscosity as the applied mixing work input increased. Increasing elongation strain resulted in an increase in the extensional stress response of the dough, suggesting a more pronounced impact of mixing on the extensional properties of dough under large deformations. Increasing *G*′ values obtained from frequency sweeps conducted in the linear region indicated increasing elasticity in doughs with continued mixing up to development peak, which was suggested to be due to protein interactions. However, small deformation shear tests were found to be difficult to provide reproducible data as they were very sensitive to dough preparation. The dough’s apparent viscosity obtained under large deformations showed a similar trend to that obtained for *G*′ under small deformations. The reproducibility of results from the large deformation shear tests was more consistent than small deformation tests in terms of determining the effect of mixing on dough rheology. Large deformation shear tests were reported to be more successful in differentiating the doughs from different wheat flours when compared to small deformation shear tests [92].

During dough mixing, flour particles are hydrated and sheared to a certain extent. As a result, they no longer exist as separate entities, as gluten proteins form a continuous network. The mechanical behavior of wheat flour dough strongly depends on the amount of water added during mixing [88]. Osorio et al. [94] used a fundamental rheological method and conducted lubricated squeezing flow tests on wheat flour doughs to determine the effect of water added at different levels on dough rheology. An optimum water absorption capacity was determined for wheat flour using the Farinograph, where the consistency reached 500 BU consistency. Moreover, the other two water levels added to wheat flour dough were ±5% of the optimum water absorption capacity. Biaxial extensional viscosity values plotted versus biaxial strain rate ($\dot{\varepsilon }$: 0.01–0.1 s^−1^) indicated a higher biaxial viscosity for the wheat flour with the lowest level of added water. This was attributed to the water level being the most restricted in this dough sample and, thus, the extent of gluten hydration being the lowest. On the other hand, extensional viscosities of doughs with optimum and excess water levels were similar, where gluten proteins were fully hydrated in both of these dough samples [94]. Strain rates experienced during dough fermentation and oven rise have been reported to be around 10^−4^ s^−1^ to 10^−3^ s^−1^, while instruments used to test doughs under extensional conditions (Brabender Extensograph and Chopin Alveograph) operate at extensional rates ranging from 10^−1^ s^−1^ to 1 s^−1^ [20]. The extensional rates applied on doughs by Osorio et al. [94] were higher than those experienced during fermentation and oven rise but similar to those applied in empirical dough testing methods that apply extension. Moreover, their findings indicated the detrimental effect of water addition below the optimum water absorption capacity of wheat flour during mixing on the resulting bread quality, as evidenced by the higher biaxial extensional viscosity of this wheat flour dough. The study by Osorio et al. [94] unraveled the impact of hydration and gluten network formation during mixing on the baking performance of wheat flour using a lubricated squeezing flow test.

The mechanical properties of wheat flour dough also greatly depend on the mixing time and the time after mixing (resting). Cuq et al. [28] evaluated the capillary flow properties of wheat flour dough as a function of mixing time (3–60 min) and rest time (0 or 120 min) after mixing. A capillary rheometer can be used if flow data at high shear rates, as in most processing operations, are needed [43]. It has been suggested to provide reliable information on shear and extensional flow properties for dough systems [28], as pressure fluctuations that are associated with slippage and dough structure sensitivity can be controlled and reduced in capillary rheometry [15,95]. Cuq et al. [28] conducted dough mixing using a Farinograph, and capillary flow properties were tested at increasing shear rates (13, 40, 133, 400, and 1333 s^−1^). The classical Bagley approach suggesting linear functions between pressure drop and L/D ratio (L/D values of the selected capillaries were 5, 10, 15, 20, 30, and 40) was used to describe the shear and extensional flow properties of wheat flour dough during mixing and resting. The effect of rest time on the capillary flow properties was found to depend on the initial mixing conditions (mixing time, mechanical energy input, etc.). A softening effect during resting was observed when the dough was mixed for short times (≤15 min), while a strengthening effect was observed when the dough was mixed for longer times (≥30 min). The softening effect was attributed to the mechanical relaxation of the gluten network that was stressed during mixing. On the other hand, the strengthening effect was attributed to chemical events and the formation of new crosslinks in the gluten network due to SS-SH interchange reactions in the presence of oxygen. The experimental Farinograph consistency correlated well (R^2^ = 0.905–0.940) with the extensional viscosities, but its correlation (R^2^ = 0.131–0.676) to the shear viscosities obtained from the capillary rheometer was poor. The exponential models suggested high correlations for the changes in the Farinograph consistency (R^2^ = 0.981) and extensional viscosity after 120 min of resting (R^2^ = 0.949) as a function of the mechanical energy input during mixing [28]. These correlations suggested the possible use of extensional viscosity obtained from capillary rheometer tests to study the changes occurring in the flow properties of wheat flour dough during mixing and during resting. Hicks and See [95] have listed the causes for pressure fluctuation that can be observed during capillary flow tests on wheat flour dough as the rupturing of large agglomerates at high shear rates, releasing of the gas entrapped in the gluten-starch matrix during mixing, inhomogeneity (due to poor mixing), and surface fracture. Therefore, it has been suggested to limit the maximum shear rate by 1000 s^−1^ in capillary rheometry tests to avoid pressure fluctuations and, thus, to obtain parameters that can be modeled to define the flow behavior of wheat flour doughs.

**Figure 6:**
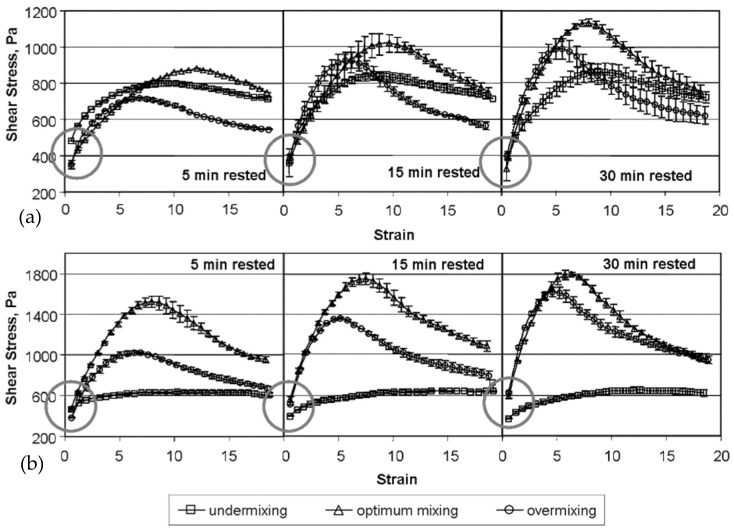
Stress responses of weak wheat flour dough (**a**) and strong wheat flour dough (**b**) during the initial 30 min of resting. Circles indicate shear stresses at small strain amplitudes. Reproduced with permission from Kim et al. [19].

Similar to Cuq et al. [28], Kim et al. [19] also studied the impact of mixing and resting on the viscoelastic properties of strong and weak hard wheat flour doughs under small and large deformations. Instead of using the capillary flow tests, Kim et al. [19] conducted stress growth tests ($\dot{\gamma }$: 0.1 s^−1^) to determine the impact of mixing and resting on the non-linear viscoelastic properties of wheat flour doughs. Strain amplitudes were calculated by multiplying the shear rate by time, and the stress growth was plotted versus the strain range of 0 to 20. On the other hand, they evaluated the linear viscoelastic properties through dynamic oscillatory shear tests (*γ*: 0.5%, *ω*: 1 Hz). The complex modulus (G*) of undermixed dough in the small deformation dynamic tests was much larger than those of optimally and overmixed doughs immediately after mixing. A decrease in G* and an increase in phase angle were found for undermixed doughs during the initial resting period (up to 30 to 45 min), while overmixed doughs showed opposite trends. These findings suggested a more elastic behavior for the undermixed doughs along with a higher viscous decay during initial resting under small deformations. G* values for optimally mixed doughs did not vary during the resting period (3 h) investigated, which were more stable for strong wheat flour (protein content: 12%, Mixograph mixing time: 5.75 min) dough compared to weak wheat flour (protein content: 11.6%, Mixograph mixing time: 2.88 min) dough. On the other hand, large deformation tests more clearly showed differences among optimal, under-, and overmixed doughs and also between doughs prepared with strong and weak flours. Optimally mixed doughs exhibited the highest peak stress and strain values for both samples, indicating higher elasticity for doughs when optimally mixed, rested, and then exposed to large deformations (Figure 6a,b). On the other hand, undermixed doughs showed a pronounced viscous-like behavior under large deformations (Figure 6a,b), pointing to the importance of gluten network formation under optimum mixing conditions to improve the mechanical properties of wheat flour dough during processing. In addition, the peak stress for the dough prepared with strong wheat flour (Figure 6b) was higher than that of the dough prepared with weak wheat flour (Figure 6a), which highlighted the resilience of the gluten network in strong wheat flour dough against the large deformations applied. The inconsistent results between small and large deformation tests implied that small and large deformation tests reflected different structural aspects of dough [19].

**Figure 7:**
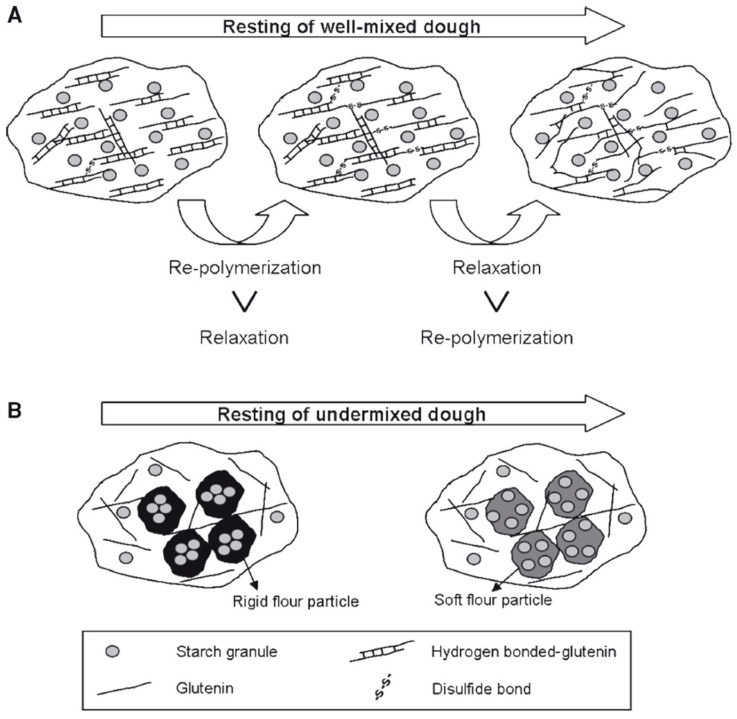
Schematic illustration of resting for an optimally developed (well-mixed) dough (**A**) and an underdeveloped (undermixed) dough (**B**). Reproduced with permission from Kim et al. [19].

The reason behind the higher G* values obtained for the undermixed wheat flour doughs under small deformation tests right after mixing was attributed to the presence of rigid flour particles in the dough due to incomplete hydration [19]. As mixing starts, water rapidly wets the outer surfaces of the flour particles. As mixing continues, the hydrated protein fibrils on the particle surfaces are wiped away by contact with the mixer blades, the sides of the bowl, or other flour particles. The resulting new particle surface is then hydrated rapidly. This is a continuous process in which flour particles are rapidly worn away, creating a continuous system of hydrated protein fibrils with starch granules dispersed throughout [58]. If mixing and hydration time are not sufficient, the resulting dough would resemble the structure of a discontinuous protein network containing a number of unhydrated flour particles composed of starch granules and unseparated proteins (Figure 7B). Therefore, the presence of rigid flour particles in undermixed dough could increase solid-like behavior in small deformation tests, as evidenced by higher G* and lower phase angle values reported by Kim et al. [19]. During resting, these rigid flour particles would become hydrated, and their rigid structure would be softened (Figure 7B). Due to the lack of developed protein structure in the undermixed dough, the hydration of flour particles during resting would directly lead to a decrease in G* and an increase in phase angle. On the other hand, the discontinuous protein network containing unhydrated flour particles in the undermixed dough would cause a number of “weak points” when the dough is exposed to large deformations. For this reason, the maximum stress of undermixed dough, which lacked a fully developed protein network, was smaller than that of optimally mixed doughs (Figure 6a,b). During the resting of an optimally mixed wheat flour dough, disulfide interchange reactions start to occur immediately after mixing, and thus, the repolymerization of glutenin is promoted. In the meantime, the stretched and aligned polymers tend to relax and return to a random orientation (Figure 7A). According to the loop-train model proposed by Belton [87] to define gluten network, HMW glutenin subunits represented by long chains have the train zones dominated by polymer–polymer interactions and the loop zones dominated by polymer–solvent interactions. Hydration of proteins, as in mixing, results in the formation of more loop regions. The stretching of the gluten network results in the deformation of the loop regions and the trains being pulled apart. When the extension is removed, the polymers relax by returning to the equilibrium of loops and trains [87,88]. However, the faster rate of disulfide interchange reactions compared to the rate of glutenin polymer relaxation results in an increase in the resistance of dough to deformations. This explains the higher stress response obtained for the optimally mixed wheat flour dough under large deformations for the different resting times studied by Kim et al. [19]. Increasing the mixing time was also reported to increase the solid-like behavior of wheat flour dough under large deformations [28]. Consequently, Kim et al. [19] showed that small deformation tests were more advantageous for understanding molecular interactions and microstructure, whereas large deformation tests were useful to evaluate practical information such as optimum mixing time and flour strength.

**Figure 8:**
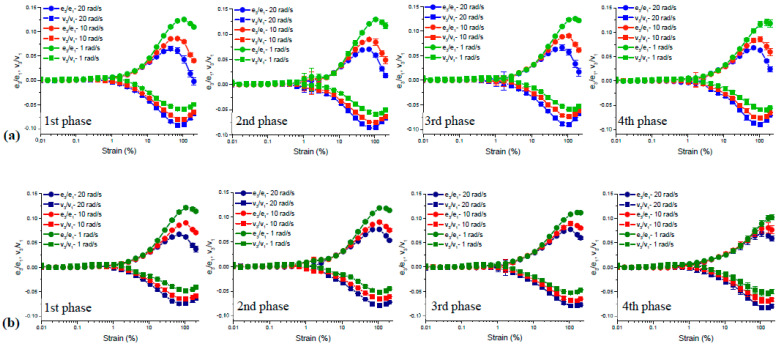
*e*_3_/*e*_1_ and *v*_3_/*v*_1_ values for soft (**a**) and hard (**b**) wheat flour doughs obtained at different stages of Farinograph mixing (*γ*: 0.01–200%, *ω*: 1, 10, 20 rad/s).

The impact of different mixing times on the non-linear viscoelastic response of hard wheat flour dough [21] and soft wheat flour dough [26] was also studied. In these studies, the LAOS technique (*γ*: 0.01–200%, *ω*: 1, 10, 20 rad/s) was used to assess the viscoelastic properties of wheat flour doughs under large deformations after being obtained at different stages of the Farinograph mixing [peak point (1st phase), indicating the development time; 5 min after the peak point (2nd phase), which shows the mixing tolerance index of the dough; 12 min after the peak point (3rd phase), which is an indicator of softening value; 20 min after the peak point (4th phase), which corresponds to the end of the Farinograph mixing measurement]. The ratio of the third-order elastic Chebyshev coefficient to the first-order (*e*_3_/*e*_1_) indicated strain stiffening behavior (*e*_3_/*e*_1_ > 0), and the ratio of the third-order viscous Chebyshev coefficient to the first-order (*v*_3_/*v*_1_) indicated shear thinning behavior (*v*_3_/*v*_1_ < 0) for both hard and soft wheat flour doughs under large deformations. The magnitude of the strain stiffening behavior started to decrease after a critical strain amplitude was reached, which was around 44–70% for soft wheat flour dough (Figure 8a) and around 110% for hard wheat flour dough (Figure 8b). These critical strain amplitudes emphasized the resilience of the strong wheat flour dough against the increasing deformations due to its higher gluten content and probably higher gluten quality (which was not mentioned in the study) when compared to soft wheat flour dough [21,26]. Because, at these critical strain amplitudes, the gluten network starts to weaken with increasing strain and the resulting mechanical energy introduced in the dough [26]. Chebyshev coefficients also showed that the gluten network had time to stretch and reached its limit in terms of its ability to elastically deform at the lower frequencies, as evidenced by the strain amplitude where the decay in the strain stiffening behavior starts to increase with the decrease in frequency (Figure 8a,b). In other words, the energy delivery from the applied strain at low frequencies is quite slow. Thus, gluten filaments find enough time to recreate network junctions that are lost during stretching—the higher rate of network junction creation than the rate of loss results in more elastic behavior [16]. A similar trend in the *e*_3_/*e*_1_ and *G_L_* values was also observed as the mixing time increased from the 1st phase to the 4th phase, suggesting a more solid-like response for the wheat flour doughs when exposed to large deformations after prolonged mixing (Figure 8a,b). The findings provided by Yazar et al. [21,26] concurred with those stated by Cuq et al. [28] and Kim et al. [19], even though different methods to assess the non-linear rheological properties of wheat flour doughs have been used in these studies. Yazar et al. [21,26] showed that the elastic component of wheat flour dough (gluten network) was affected by mixing more than the viscous component (starch matrix) because the changes occurring in the LAOS data for the elastic component became more pronounced as mixing proceeded. Therefore, Bonilla et al. [96] focused on the changes occurring in the gluten network in wheat flour dough systems during mixing and brought new insights by coupling the quantum dot labeling and Confocal Laser Scanning Microscopy (CLSM) with the strain sweeps (*γ*: 0.01–200%, *ω*: 1 rad/s).

For instance, the raw *G*′ value obtained for the soft wheat flour dough obtained at the peak time of the Farinograph mixing was 1002 Pa in the non-linear viscoelastic region (*γ*: 200%), while it was 699 Pa for the same dough obtained 10 min after the peak. These *G*′ values obtained under large deformations from the oscillatory shear tests were consistent with the consistency data obtained from Farinograph mixing, as the consistency at the peak was 450 BU, and it was 350 BU 10 min after the peak. During this 10-min Farinograph mixing of soft wheat flour dough (from peak to 10 min after the peak), the co-localization coefficients obtained from the imaging software decreased for the interactions between gliadin and HMW glutenins. The lower co-localization coefficients indicated partially mixed gliadin with separate HMW-glutenin agglomerates. Moreover, these HMW-glutenin agglomerates were suggested to be responsible for the decay in dough consistency during mixing [96]. The agglomeration of HMW-glutenin subunits was reported to play a significant role in the network disruption of hard wheat flour dough in the later stages of Farinograph mixing, where the consistency was again below 500 BU [97]. The loss of adhesion between polymers or particles is known as the Payne effect in material science. The breakdown of particle–particle interactions or the detachment of particles from polymers causes a reduction of moduli through the dissipation of energy by the facilitated sliding of polymer chains/particles [98], which explains the decrease in *G*′ for soft wheat flour dough during the Farinograph mixing of peak to 10 min after the peak [96]. Apart from the interactions between gliadins and HMW-glutenins, Bonilla et al. [96] also evaluated the interactions between gliadins and LMW-glutenins through the PNA software and reported a decrease in the area and in the number of junctions for the gliadin-LMW-glutenin network when the dough was mixed from peak to 10 min after the peak. These findings suggested a breakdown in the gluten network during the mixing of soft wheat flour with 8% protein. On the other hand, hard wheat flour dough showed a consistency decay of 80 BU, and durum wheat flour dough showed a 30 BU decrease when mixing continued 10 more minutes after the peak. However, *G*′ values (*γ*: 0.01–200%) for doughs mixed until peak time and 10 min after peak overlapped for both hard wheat flour (11% protein) and durum wheat flour (13.2% protein). The co-localization coefficient for gliadin and LMW-glutenins increased as mixing proceeded from peak time to 10 min after the peak, while the co-localization of gliadin and HMW-glutenin did not change significantly. This suggested that the mobility of gliadins and LMW-glutenins contributed to the stability of the gluten network in hard wheat flour dough with prolonged mixing [96]. The similar *G*′ values recorded up to the strain amplitude of 200% and the in-situ visualization of increasing gluten strength for hard wheat flour dough as mixing time increased [96] could be attributed to the redistribution of gliadins and LMW-glutenins after mixing, as suggested by others [19,28]. The interactions between gliadins and LMW-glutenins after mixing until rheological testing and image analysis might have compensated for the decay observed in the Farinograph consistency (80 BU). Finally, no significant changes were found when analyzing the network parameters of each subunit at peak and 10 min after peak for durum wheat flour dough. The stability of Farinograph consistency, *G*′ values up to 200% strain, and the overlapped images obtained from in-situ visualization for durum wheat flour dough at different mixing times collectively pointed to the stability of the gluten network in durum wheat flour dough against the mixing forces. This higher stability in durum wheat flour dough was attributed to the higher ratio of LMW to HMW-glutenins when compared to soft and hard wheat flour doughs [96]. During wheat flour dough mixing, LMW-glutenins were found to detach from the HMW-glutenin backbone through the disruption exerted by mechanical forces. In the presence of excessive LMW-glutenins in wheat flour, as in the case of durum wheat flour, other LMW-glutenins can replace the detached LMW-glutenins. Thus, the gaps in the gluten network can be constantly filled, leading to a more stable gluten network [97]. Consequently, by coupling oscillatory shear tests with the image analyses used for in-situ detection of the changes occurring in wheat flour dough during mixing, Bonilla et al. [96] provided a quantitative analysis of how the distributions of gluten fractions impacted the stability of wheat flour doughs against the varying mixing times.

**Figure 9:**
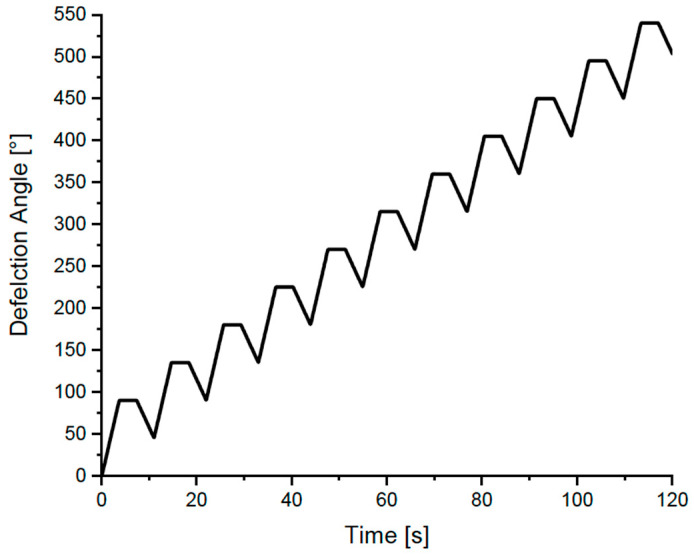
The rheological method based on increasing deflection used to mimic the deformations applied in a shear kneading process. Reproduced with permission from Vidal et al. [32].

The studies discussed above pointed out changes occurring in dough rheology during the resting period after mixing until the rheological testing. To eliminate the issues due to sample transfer between the kneader and a measurement device, Vidal et al. [22] used a conventional rheometer to apply an in-line measurement setup based on a shear kneading process. The protocol they used consisted of the application of oscillatory shear deformation with an asymmetric deflection angle back and forth. After each 90° forward deformation, a 4-s relaxation step was implemented, which was followed by a 45° reverse deformation, as shown in Figure 9. In this setup, a plane plate–cylinder geometry was used to enable dough formation during testing. The network evolution during this shear-induced kneading in the rheometer was evaluated by fitting the relaxation modulus after each kneading step using the following equation:

$$G\left(t\right)=\frac{\sigma (t)}{\gamma_{0}}=St^{-r}$$

where *G*(*t*) is the linear relaxation modulus, *σ*(*t*) is the shear stress at time *t*, *γ*_0_ is the shear strain at the beginning of the relaxation, *S* is the stiffness of the matter, and *r* is the relaxation exponent. A decrease in the *S* values was recorded after 150 s of shear kneading, which corresponded to the dough development time reached in the DoughLAB mixing, suggesting a decrease in the gel strength beyond this point of mixing. As evidenced by the *S* values, the shear kneading setup successfully produced an optimally developed dough matrix close to the reference kneading time of 150 ± 7.9 s [22]. Stress relaxation measurements conducted at small strain amplitudes (0.1%) for different doughs with different strengths showed no difference in the relaxation behaviors of the tested doughs [99]. However, at large strains (>29%), the relaxation behavior was found to better correlate with the strength of the dough [31]. The large deformations applied in the stress-relaxation kneading, with a set strain amplitude of 650% during the process, were suggested to be sufficient to determine the dough strength under mixing deformations. Therefore, the shear kneading protocol proposed by Vidal et al. [32] could be useful as an in-line tool to quantitatively determine the changes occurring in the gluten-starch matrix during mixing, which might replace the recording mixers based on empirical testing.

### 4.3. Fermentation (Proofing) and Baking Quality

Although mixing is the most critical and complex operation in breadmaking, proofing is still the heart of breadmaking that links the air bubbles incorporated in the gluten–starch matrix during mixing to the gas cell distribution in the baked loaf [46]. Wang and Sun [33] conducted a non-linear creep recovery test as a fundamental method and the empirical dough testing methods of the Farinograph, Mixograph, and Kieffer dough extensibility tests to predict the baking performances of 23 different wheat flours. The maximum creep strains obtained from the creep recovery tests [creep phase: *F* = 50 mN (*σ* ≅ 636 N/m^2^), *t* = 4 min; recovery phase: *F* = 5 mN, *t* = 4 min; T = 25 °C] for wheat flour doughs (54% added water on flour basis, *v*/*w*) did not show an apparent correlation with loaf volume (r = 0.122). However, the maximum recovery strains were positively correlated to bread loaf volumes (r = 0.939). This correlation suggested that wheat flour dough should show a high recovery strain to produce bread with a high loaf volume. The recovery strain was shown to indicate dough elasticity; thus, it was suggested to be an important parameter for dough film stability. The higher the recovery strain, the better the stability against the rupture of dough films between gas cells. The elasticity of the gluten–starch matrix surrounding the gas cells should be high enough to prevent CO_2(g)_ release from the dough. The maximum recovery strain was also found to be positively correlated with resistance to extension (r = 0.947) from the Kieffer test, Mixograph mixing time (r = 0.889), and Farinograph water absorption (r = 0.736). Thus, this study suggested the use of maximum recovery strain as a fundamental parameter to classify and predict flour quality for breadmaking, which could be obtained by testing a small amount of dough as low as 0.5 g [33]. Van Bockstaele et al. [34] also used the non-linear creep recovery tests (creep phase: *σ* = 100 Pa and 250 Pa, *t* = 5 min; recovery phase: *σ* = 0 Pa, *t* = 10 min; T = 20 °C) to determine the baking quality of wheat flours from 17 pure wheat cultivars. The data obtained were evaluated using the Burger’s model. The parameter *r*_2_ (retardation time) was found to be an interesting parameter that might contain information regarding the viscoelastic properties related to baking quality. Using this parameter along with the steady-state viscosity (*μ_0_*), it was possible to categorize the wheat flours studied based on their baking performances. Wheat flours with the lowest *r*_2_ values (ranging from 52.5 ± 0.6 s to 59 ± 0.6 s) had the lowest loaf volumes in the resulting bread (429 ± 7 to 557 ± 9 cm^3^/100 g flour), while wheat flours with high *r*_2_ values (ranging from 61.1 ± 0.3 s to 65.2 ± 0.2 s) showed the highest bread loaf volumes (506 ± 12 to 641 ± 5 cm^3^/100 g flour). These findings suggested that higher loaf volumes could be obtained from wheat flour doughs with a higher retardation time (*r*_2_), indicating a slower recovery in the non-linear creep recovery tests [34]. In contrast, Kawai et al. [100] reported an inverse relationship between the retardation time and bread volume. However, only three wheat cultivars with different water contents were tested in their study. The decrease in the retardation time (*r*_2_) was associated with the weakening of the elastic restoring forces, causing the dough to flow more readily. Another reason for this controversy could be due to the differences in the strengths of the wheat flour tested. For example, if the wheat flours tested by Kawai et al. [100] had higher gluten content and quality than those tested by Van Bockstaele et al. [34], the reduction in *r_2_* might have contributed to loaf volume. Meerts et al. [51] observed a decrease in the retardation time as the water content in wheat flour dough was increased. This finding indicated that the elastic deformation recovered much faster at higher water levels. Thus, it could be concluded that the positive correlation between *r*_2_ and loaf volume, as suggested by Van Bockstaele et al. [34], could be due to the elastic component dominating the viscoelastic properties of wheat flour dough. Rouillé et al. [24] conducted non-linear creep recovery tests and lubricated squeezing flow tests to determine the impact of minor wheat flour components (water-soluble components, puroindolines, lipids) on shear and biaxial extensional properties of wheat flour doughs. Both tests (T = 20 °C) indicated a shear thinning behavior for wheat flour dough at higher shear rates up to 1 s^−1^ and pointed to the lubricating effect of the water-soluble fraction on the non-linear viscoelastic properties of wheat flour dough. The comparison between shear and extensional properties through the Trouton number indicated that the lubricated squeezing flow test was more efficient in terms of discriminating between wheat flours from the same cultivar when compared to creep recovery tests [24]. Trouton number (*N_T_*) is a dimensionless number that is used to compare the relative magnitude of extensional (*η_E_*, *η_B_* or *η_P_*) and shear (*η*) viscosities [43]:

$$N_{T}=\frac{extensional\;viscosity}{shear\;viscosity}$$

The Trouton ratio for a Newtonian fluid is 6 in biaxial extension [43]. The Trouton number for the insoluble fraction of wheat flour was 20, while it was 10 for the reconstituted dough of soluble and insoluble fractions. Thus, the increase in Trouton number was related to the elasticity of wheat flour dough and pointed out the lubricating effect of the soluble fraction. In this study, the loaf volumes of the resulting breads were found to be inversely related to biaxial extensional viscosity (*η_B_*) at $\dot{\varepsilon }$*_B_* = 10^−1^ s^−1^ [24]. Ktenioudaki et al. [101] also reported a negative relationship between biaxial extensional viscosity and loaf volume (r = −0.8, −0.8, −0.7, −0.7, and −0.6 for strains of 0.1, 0.25, 0.5, 0.75, and 0.95, respectively). Similarly, a negative relationship was found between biaxial extensional viscosity and dough height during proofing as measured by the Rheofermentograph (r = −0.8, *ε_B_* = 0.5, $\dot{\varepsilon }$*_B_* = 0.01 s^−1^). These negative relationships suggested that high biaxial extensional viscosity limited dough rising during proofing or oven-rise [101]. On the other hand, biaxial extensional viscosity obtained from lubricated squeezing flow tests was found to control the homogeneity of dough cellular structure during fermentation, which was attributed to the resistance it imparted to the gluten–starch matrix against the coalescence of the expanding gas cells [29]. The increase in extensional viscosity with increasing biaxial strain could be defined as the strain hardening index [7,102]. Therefore, an increase in biaxial viscosity during proofing and oven rise is required for a homogeneous expansion of the gas cells, but it should not be too high, similar to the strain stiffening behavior as suggested by Yazar et al. [22]. The extensional viscosity of wheat flour doughs was reported to increase with increasing protein content in wheat flour. Thus, the possibility of preparing different wheat flour doughs by manipulating the protein content was indicated to maintain the desired extensional properties required to process specific baked foods, such as bread, cookies, cake, etc. [43].

The strain hardening index (*SHI*) is another parameter obtained from lubricated squeezing flow tests to evaluate the extensional properties of wheat flour doughs. It can be calculated using the parameters stress and extensional deformation. For this purpose, the stress (*σ*) values obtained from extensional tests at a given extensional strain rate are ($\dot{\varepsilon }$) plotted versus deformation (*ε*) on a logarithmic scale. Furthermore, the slope of this curve is defined as the strain hardening index [24,29,30]. *SHI* determined by the lubricated squeezing flow tests was found to play a crucial role in determining crumb fineness, as evidenced by the correlation (r^2^ = 0.83) between these parameters. Higher strain hardening was suggested to limit bubble coalescence in the dough, thus leading to a higher proportion of finer cells in the crumb [24]. Fine crumb structures with a large number of small size and thin-walled cells were reported to be among the good baking quality characteristics [101]. Homogeneous growth of gas bubbles in the dough without premature rupture and coalescence should favor maximum loaf volume development [102]. The resistance of the dough wall between two adjoining gas cells against expansion depends on the strain-hardening behavior of the gluten–starch matrix, which directly influences the rupture and coalescence of the expanding gas cells. Thus, Ktenioudaki et al. [101] reported a good correlation between *SHI* and loaf volume (r = 0.7), where the baking quality of different wheat varieties was studied. However, instead of *SHI*, they suggested the biaxial extensional viscosity obtained from lubricated squeezing flow tests to be a critical parameter in determining loaf volume, especially when coupled with the extensibility obtained from the empirical Extensograph (uniaxial extension) test [101]. Simultaneous evaluation of several rheological properties, such as strain hardening, resistance to extension, and extensibility, was reported to better predict baking quality rather than focusing on a single parameter [102]. Higher uniaxial extensibility and lower biaxial extensional viscosity in wheat flour doughs were found to result in bread with higher loaf volumes [103]. In another study evaluating the baking quality of different wheat varieties, Ktenioudaki et al. [103] reported a poor correlation (r = 0.5) between the strain hardening index obtained from lubricated squeezing flow tests for wheat flour doughs and the loaf volume of the resulting bread. This poor correlation, conflicting with the correlation found by Ktenioudaki et al. [101], was attributed to the small variation observed in the baking volume of the varieties—because the difference in strain hardening index was pronounced for the weakest (*SHI* = 1.3, loaf volume = 168 mL) and strongest (*SHI* = 2, loaf volume = 180–190 mL) wheat varieties [103]. For a good baking performance, *SHI* in biaxial extension was suggested to be larger than 2, assuming a constant strain rate during oven rise and baking [54,55], while Dobrazczyk and Roberts [104] suggested values larger than 1. On the other hand, the *e*_3_/*e*_1_ (indicative of strain stiffening when >0) obtained from LAOS deformation showed a maximum between 0.1–0.2, which was followed by a decrease as strain amplitude increased [21,26]. A constant increase in *e*_3_*/e*_1_ versus increasing shear deformation was found to be detrimental to loaf volume [52]. It should be noted that the strain stiffening values defined for wheat flour doughs were dependent on the nature of the applied deformation and the geometry used.

**Figure 10:**
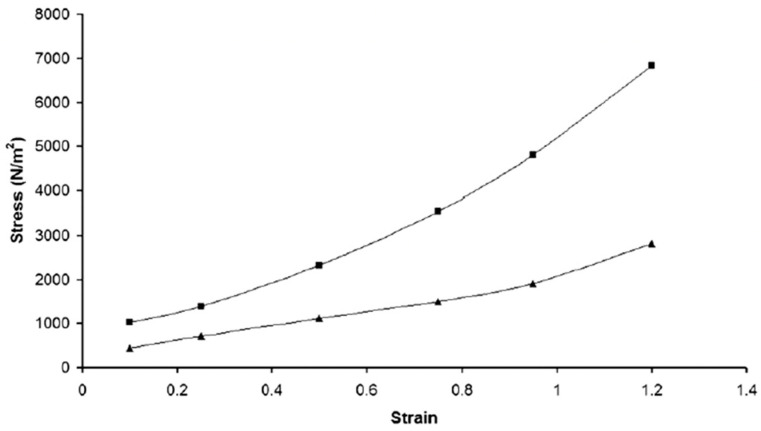
Stress as a function of strain in uniaxial and biaxial extensions at a constant strain rate of 0.01 s^−1^ for wheat flour dough (T = 32 ± 2 °C): 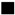 uniaxial extension, 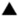 biaxial extension. Reproduced with permission from Ktenioudaki et al. [103].

The variation in potential for gas retention among wheat flour doughs was mainly attributed to differences in the large-deformation properties of dough films [7,54,55]. Therefore, the uniaxial and biaxial extensional properties of wheat flour doughs have been mostly studied to predict their gas retention capabilities during proofing. Sliwinski et al. [47] conducted small deformation and large deformation tests, which were biaxial extension (uniaxial compression) and uniaxial extension (Kieffer rig) tests (T = 25 °C), on wheat flour dough to evaluate the processing qualities of wheat flours from different cultivars. The force-displacement curves obtained from the biaxial extension and uniaxial extension tests were recalculated into stress-strain curves assuming a constant volume for the dough sample at a constant strain rate of 0.01 s^−1^, as shown in Figure 10. For all wheat flour doughs, the uniaxial and biaxial stresses increased more than proportionally with the strain, which was indicative of strain stiffening. Higher stresses in uniaxial than in biaxial extension were reported for the same wheat flour dough (Figure 10), which was attributed to the orientation of the network elements in one direction when the dough was exposed to uniaxial extension. However, in biaxial extension, the dough is extended both in the direction of the extension and in the direction perpendicular to the extension. Therefore, to prevent anisotropy of dough films in the baking process, it is preferable to promote a biaxial structure in the dough. For this purpose, sheeting and lamination have been used in the baking industry as part of the process for the production of cookies, pizza, bread, and pastry dough. These processing steps have been suggested to develop a biaxial structure in the dough by combining the sheeting action of the rollers with rotation of the axis of the dough between successive passages. Apart from the differences between uniaxial and biaxial extension properties of wheat flour doughs from different wheat cultivars, Sliwinski et al. [47] found more pronounced differences between these wheat flour doughs using large deformation tests when compared to small deformation tests. The results found by Ktenioudaki et al. [103] concurred with the findings of Sliwinski et al. [47], as the stress during uniaxial extension was higher than that measured during biaxial extension at all strains studied, and the difference between the uniaxial and biaxial stresses increased with increasing strain (Figure 10).

Considering that the empirical dough testing methods that apply biaxial extension, such as the Alveograph, have been proven to provide useful data to predict the breadmaking quality of wheat flours [15,43], Dobraszczyk et al. [105] improved the dough inflation method using a texture analyzer working at a constant strain rate. This dough inflation method allowed the determination of rheological properties such as stress, strain, biaxial viscosity, and extensional strain hardening values, which were used to discriminate the baking quality of commercial wheat flours [24,106].

Dobraszczyk and Salmanowicz [107] conducted three different large deformation tests (Kieffer dough extensibility, D/R dough inflation, and Mixograph tests) on wheat flour dough to predict its baking quality. Among the parameters obtained from these tests, bubble failure strain (R = 0.881) and *SHI* (R = 0.855) measured at 50 °C using the dough inflation method provided the highest correlations with baking volume. The magnitudes of the applied deformation were not defined in the Mixograph and Kieffer dough extensibility tests. However, the parameters obtained from the dough inflation test were extracted from the strain-stress curves. Thus, this study indicated a better baking quality prediction using a fundamental approach where the applied strain and the resulting stress (or vice versa) were defined instead of using parameters with arbitrary units. These findings also indicated that biaxial deformations applied on wheat flour dough, as in dough inflation tests, better approximated the deformations of the breadmaking process when compared to uniaxial extension applied in the Kieffer dough extensibility test and to complex deformations applied in the Mixograph.

It has been suggested that extensional rheological properties of doughs at elevated temperatures (50 °C) were sensitive indicators of changes in the secondary structure of the gluten polymer and could be used as predictors of baking quality. Conducting extensional tests at 50 °C was found to provide the best discrimination between wheat flours of varying baking quality [107,108]. This could be due to the strong increase in the strain-stiffening behavior of wheat flour doughs up to 50 °C during heating. With progressive heat treatment, a decrease in the strength of hydrogen bonds has been suggested, which limits the ability of the gluten strands to interact via short-range interactions. As a result, the slippage of proteins along each other without intermediate stabilization has been considered to cause strain stiffening. The initiation of conformational changes causes the aggregation of gluten proteins and changes the ability of the loop regions to extend upon elongation. Thus, reaching the extensibility limit might explain the triggering of strain stiffening at the early stages of baking. On the other hand, a decrease was reported in the strain stiffening index of wheat flour dough between the temperatures of 50 °C and 70 °C [30], which indicated that the temperature of 50 °C was a critical temperature in terms of strain stiffening behavior. However, as discussed above, most of the studies evaluating the extensional properties of wheat flour doughs to predict baking quality have been conducted at around 30 °C, except for the study conducted by Dobraszczyk and Salmanowicz [107].

**Figure 11:**
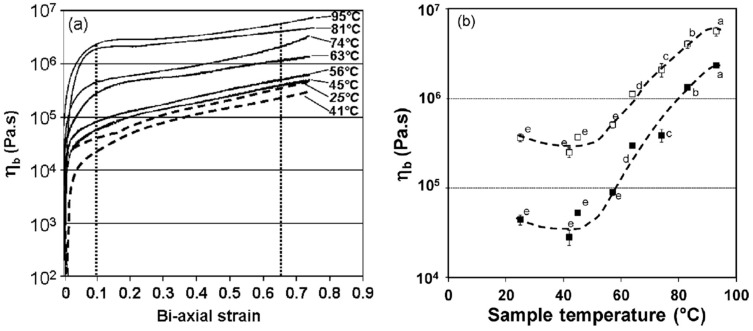
Effect of temperature on the biaxial viscosity of wheat flour dough measured during lubricated squeezing flow tests: (**a**) biaxial viscosity versus biaxial strain at different temperatures, (**b**) biaxial viscosity versus temperature at biaxial strains of 0.1 (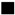) and 0.65 (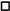), where different letters for the same response indicate significant differences between data points (*p* ≤ 0.05). Reproduced with permission from Vanin et al. [7].

Recent studies have focused on the application of increasing temperature at large strains to characterize the rheological properties of wheat flour dough under conditions that are as close as possible to the real baking process. Vanin et al. [7] conducted lubricated squeezing flow tests ($\dot{\varepsilon }$*_B_* = 0.005 s^−1^, *ε_B_* = 0.1 and 0.65) on wheat flour dough at temperatures ranging from 25 °C to 95 °C. At both strains, the extensional viscosity of wheat flour dough decreased as the temperature increased from 25 °C to 40 °C (Figure 11a), which was described by the Arrhenius equation. After reaching a minimum at 40 °C, biaxial viscosity increased dramatically as the temperature increased above 50 °C (Figure 11a). The difference in viscosity at different strains was much larger in the temperature range of 25–45 °C than in the range of 60–95 °C (Figure 11b), suggesting a higher degree of strain stiffening behavior for wheat flour dough below 60 °C than above [7]. This finding concurred with the findings of others [30,107,108] and supported the possibility of better predicting the baking quality of wheat flours when extensional tests were conducted below 60 °C.

**Figure 12:**
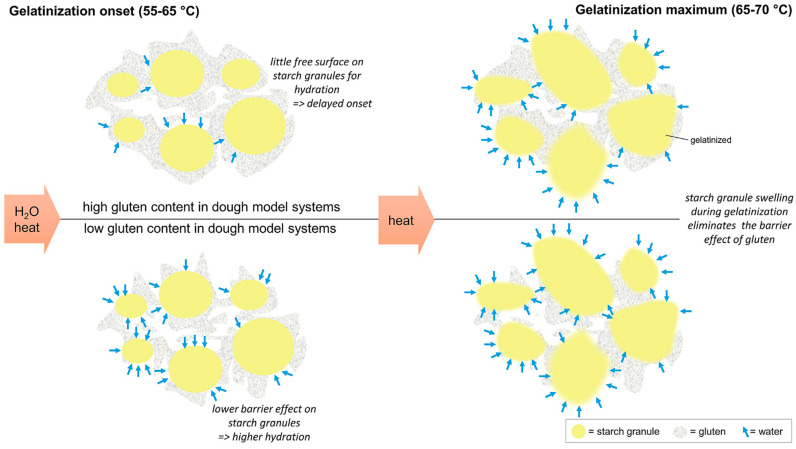
Effect of gluten content in wheat flour on starch gelatinization onset during heating. Reproduced with permission from Jekle et al. [109].

Launay and Michon [102] suggested conducting biaxial extension tests followed by stress relaxation to predict the baking quality of wheat flours, as the stress relaxation properties of doughs were related to their processing behavior. Vanin et al. [7] conducted stress relaxation tests *(ε_B_*_,*max*_ = 0.8, *t* = 180 s) after lubricated squeezing flow tests at temperatures ranging from 25 °C to 95 °C. The relaxation degree remained constant (98–99%) at low temperatures. However, it started to decrease significantly above 56 °C, suggesting an onset for the transition from viscoelastic dough into a solid-like crumb due to a series of physicochemical changes occurring in starch and gluten proteins (i.e., starch gelatinization and gluten network thermosetting). The onset of starch gelatinization during baking was reported to depend on the gluten content in wheat flour [109]. In the presence of high gluten content in wheat flour, the hydration of starch granules was suggested to be hindered, and thus, the onset of starch gelatinization shifted to higher temperatures (Figure 12). Therefore, it should be noted that the temperature at which the relaxation degree starts to decrease might be different depending on the gluten content of the wheat flour being tested when the dough was biaxially deformed at different temperatures prior to stress relaxation as performed by Vanin et al. [7].

Alpers et al. [30] also used lubricated squeezing flow tests to determine the baking quality of wheat flour with an in-line fermentation and baking method. In this setup, dough samples were first fermented at 30 °C for 60 min. During fermentation, the dough was allowed to expand uniaxially by adjusting the upper plate to keep the normal force at a constant level of 10 g. A silicone jacket (0.5 mm, Sahltec, Bremen, Germany) and a customized 3D-printed shell (GreenTec Pro, Extruder FD3D GmbH, Lauterach, Austria) were used to achieve uniaxial extension. The in-line fermentation step was followed by the heat treatment controlled by an external thermometer. After reaching the selected baking temperatures of 30 °C, 50 °C, 60 °C, 70 °C, 80 °C, and 85 °C the silicon jacket and the 3D-printed shell were removed to allow a free squeezing flow (biaxial extension) of the dough. The biaxial viscosity (*η_B_*) results ($\dot{\varepsilon }$*_B_* = 1 s^−1^) for non-yeasted dough indicated that the most significant conformational changes in gluten occurred due to fewer hydrophobic interactions within the temperature range of 50 °C to 70 °C, concurring with the findings of Vanin et al. [7] at $\dot{\varepsilon }$*_B_* = 0.65 s^−1^. At temperatures above 70 °C, only minor changes were detected. On the other hand, the *η_B_* for yeasted dough showed a further increase beyond 70 °C until the final baking temperature, which was attributed to the polymerization of gluten proteins initiated at temperatures above 70 °C. The pre-extended gluten strands of the yeasted dough system were suggested to cause an earlier onset of strain hardening, leading to an overall increase of the *SHI* for yeasted dough systems. The changes probed between the biaxial viscosities of these two dough systems were more pronounced at higher biaxial extension rates [30]. Extensional rheology was suggested to determine gluten functionality in wheat flour doughs by mainly addressing long-range interactions [29]. Thus, the changes in the *η_B_* were mainly associated with the changes in gluten structure [30].

Most of the recent techniques involved the application of biaxial extension to determine the fermentation and baking quality of wheat flour. As another alternative, Vidal et al. [110] proposed a rheo-baking method to characterize crumb evolution and volume expansion in wheat flour doughs produced with different leavening agents. In this method, the settings used for the rheo-kneading [32] were used. Dough samples were held at 30 °C at 80% *RH* for 30 min with an applied normal force of 1 N to imitate the proofing step. Then, the temperature was increased with a ramp of 4 °C/min, which was determined according to the temperature rise in a baked product in a deck oven until reaching 95 °C. The baking quality of wheat flour in the presence of different leavening agents was determined through the change in the rheometer gap (*h*) using the equation for the volume of a cylinder (*V* = $\pi \;r^{2}\;h$). The results obtained from rheo-baking showed high comparability to those of standard baking tests. Thus, the rheo-baking method was suggested to be a useful tool to analyze the baking performance of wheat flour as well as the functionality of ingredients with a wheat flour expenditure as low as 384 mg [110]. Even though this technique does not involve the application of large deformations on dough samples, it can be considered as an alternative to the non-linear rheological testing methods as it allows the dough to deform and transform from a viscoelastic system into a solid-like aerated system simulating the proofing and baking steps under a defined geometry and a controlled testing protocol.

All the studies discussed above revealed how wheat flour quality could alter the viscoelastic properties of wheat flour doughs under large deformations and, thus, the textural and sensory attributes of the resulting baked products. Recently, Cyriac et al. [111] proposed developing multivariate machine learning models, where several key instrumental textural attributes could be predicted using the data obtained from the SAOS and LAOS tests. This method could be another alternative to determine the baking performances of wheat flours with different quality characteristics.

The fundamental non-linear rheological methods used for determining wheat flour quality in terms of gluten quality and quantity, mixing properties, fermentation, and baking performances are summarized in Table 2.

## 5. Conclusions

Fundamental rheological tests conducted under large deformations were shown to differentiate between wheat flours based on their processing capabilities. Among the fundamental methods, uniaxial extension, stress relaxation, and LAOS tests were commonly used to characterize wheat flour doughs based on gluten quality and quantity and characterize the viscoelastic response of gluten and gluten fractions under large deformations. The methods used for studying the mechanical response of wheat flour doughs during mixing included extrusion tests, capillary flow tests, stress growth tests, LAOS tests, and stress relaxation tests conducted with a change in the deflection angle between shear deformation and relaxation. The reason behind the diverse deformations applied through these methods was to approach the complex deformations involved in dough mixing. The coupling of these methods with imaging techniques was suggested to bring a more detailed characterization of the mixing behavior of wheat flour doughs. On the other hand, fundamental methods based on biaxial extensional deformation were mostly preferred to study the non-linear viscoelastic response of wheat flour doughs during proofing and oven rise. For this purpose, lubricated squeezing flow tests were commonly used. As a recent alternative, the application of biaxial extensional tests followed by stress relaxation was suggested to better characterize the non-linear viscoelastic properties of wheat flour dough during proofing and oven rise. Another recent technique involved a special geometry setting, allowing the dough to rise uniaxially as the temperature increased with a certain ramp to evaluate the changes occurring in wheat flour dough rheology during baking.

Most of the studies evaluating wheat flour quality using large deformation fundamental rheological tests have focused on protein content, mixing properties, fermentation, and the baking capabilities of wheat flour. This review revealed a gap in the literature regarding the evaluation of gluten index, sedimentation, or falling number values of wheat flours using the non-linear fundamental rheological testing methods. Further studies can be conducted to determine the impact of milling on wheat flour baking quality by characterizing the non-linear viscoelastic properties of wheat flour doughs from wheat flours with varying damaged starch content or yield. Thus, a solid link between wheat flour quality and baking performance can be built through the use of fundamental rheological testing methods conducted under large deformations.

Future studies should focus on improving the recently used techniques discussed in this review to assess wheat flour quality based on baking performance. As a recently emerging fundamental non-linear rheological method, the LAOS technique can be used to evaluate the non-linear rheological properties of wheat flours from different varieties. The correlation between the LAOS parameters and the baking test results or biaxial extensional deformation test results can be evaluated considering the Trouton number. LAOS and SAOS tests can be coupled (stop-flow amplitude-frequency sweeps) to unravel the changes occurring in the microstructure of the gluten-starch matrix under different magnitudes of deformations. The data obtained from SAOS and LAOS tests for wheat flour doughs can be used to develop multivariate machine learning models that enable the prediction of several key instrumental textural attributes of the resulting products. Moreover, non-linear creep recovery tests conducted on wheat flour doughs can be coupled with 3D-printing technology to improve baked product quality, considering the raw material quality. Thus, new fundamental rheological dough testing methods can be developed for the quality assessment of wheat flours based on baking performance, with the possibility to obtain more detailed and accurate data than those obtained with empirical dough testing methods.

## Figures and Tables

**Table 2:** Fundamental rheological methods used for wheat flour quality characterization.

Wheat Flour Quality Parameter	Dough Sample Tested	Non-Linear Fundamental Rheological Method
Gluten quality and quantity	Wheat flour dough	Uniaxial extension test ^a^
	Gluten	Uniaxial extension test ^b^
	Hard and soft wheat flour dough	LAOS test ^c^
	Soft wheat, hard wheat, and durum wheat flour doughs	LAOS test ^d^
	Gluten	LAOS test ^e^
	Gluten, with and without endogenous lipids	LAOS test ^f^
	Defatted gluten	Stress relaxation test ^b,g^
	Gliadin and glutenin	Stress relaxation test ^g^
	Gliadin and glutenin	LAOS test ^h^
Mixing behavior	Hard and soft wheat flour doughs (exposed to different levels of mixing energy input)	Extrusion test, planar extensional test, large deformation shear test ^i^
	Wheat flour dough (with different levels of added water)	Lubricated squeezing flow test ^j^
	Wheat flour dough (at different mixing and resting times)	Capillary flow test ^k^
	Strong and weak wheat flour doughs (at different mixing and resting times)	Stress growth test ^l^
	Hard and soft wheat flour doughs (obtained at different stages of Farinograph mixing)	LAOS test ^c^
	Soft wheat, hard wheat, and durum wheat flour doughs (obtained at different stages of Farinograph mixing)	Strain sweep test coupled with quantum dot labeling and CSLM ^m^
	Wheat flour dough	Stress relaxation test (with a change in the deflection angle between shear deformation and relaxation) ^n^
Fermentation and baking performances	Wheat flour dough (from different wheat cultivars)	Creep recovery test ^o,p^
	Wheat flour dough and reconstituted dough	Creep recovery test ^q^ Lubricated squeezing flow test ^q^
	Wheat flour dough (from the wheat of different regions)	Lubricated squeezing flow test ^r^
	Wheat flour dough (with different formulations and different mixing procedures)	Lubricated squeezing flow test ^s^
	Wheat flour dough (from different varieties of wheat)	Lubricated squeezing flow test ^t^
	Wheat flour dough (from different wheat cultivars)	Biaxial extension (uniaxial compression) test ^u^
	Wheat flour dough	D/R inflation method (T = 50 °C) ^v^
	Wheat flour dough	Lubricated squeezing flow test (T = 25–95 °C) followed by stress-relaxation test ^w^
	Wheat flour dough	Lubricated squeezing flow test at select temperatures (in-line fermentation and baking test) ^x^
	Wheat flour dough	Rheo-baking (controlled fermentation and baking under defined geometry) ^y^

^a^ Uthayakumaran et al. [8], ^b^ Uthayakumaran et al. [80], ^c^ Yazar et al. [21,26], ^d^ Erturk et al. [18], ^e^ Ng et al. [86], ^f^ Yazar et al. [22], ^g^ Li et al. [31], ^h^ Yazar et al. [90], ^i^ Zheng et al. [92], ^j^ Osorio et al. [94], ^k^ Cuq et al. [28], ^l^ Kim et al. [19], ^m^ Bonilla et al. [97], ^n^ Vidal et al. [32], ^o^ Wang and Sun [33], ^p^ Van Bockstaele et al. [34], ^q^ Rouillé et al. [24], ^r^ Ktenioudaki et al. [101], ^s^ Turbin-Orger et al. [29], ^t^ Ktenioudaki et al. [103], ^u^ Sliwinski et al. [47], ^v^ Dobraszczyk and Salmanowicz [107], ^w^ Vanin et al. [7], ^x^ Alpers et al. [30], ^y^ Vidal et al. [110].

## Data Availability

Data is contained within the article.

## Nomenclature

BU	Brabender unit (unit of consistency in the Farinograph test)
*e*_3_/*e*_1_	ratio of the third-order elastic Chebyshev coefficient to the first-order elastic Chebyshev coefficient (-) (from the LAOS tests)
*ε*	biaxial strain (-)
$\dot{\varepsilon }$	biaxial strain rate (s^−1^)
*F*	force (N)
*G*′	elastic modulus (Pa)
*G**	complex modulus (Pa)
*G*′*_M_*	minimum strain modulus (Pa) (from the LAOS tests)
*G*′*_L_*	large strain modulus (Pa) (from the LAOS tests)
*G(t*)	stress relaxation modulus (Pa) (from stress relaxation tests)
*γ*	shear strain (%, -)
$\dot{\gamma }$	shear strain rate (s^−1^)
h	height of a cylinder (mm) (the gap between the plate-cylinder geometry in rheo-baking tests)
*I*_3_/*I*_1_	the ratio of the third harmonic intensity to the first harmonic intensity (-) (from the LAOS tests to evaluate the MAOS region)
*I*_2_ */I*_3_	the ratio of the second harmonic intensity to the third harmonic intensity (-) (from the LAOS tests to evaluate the MAOS region)
*I*_5_ */I*_3_	the ratio of the fifth harmonic intensity to the third harmonic intensity (-) (from the LAOS tests to evaluate the MAOS region)
LAOS	Large Amplitude Oscillatory Shear
MAOS	Medium Amplitude Oscillatory Shear
MS	mixing stability
MT	mixing time
*η*	shear viscosity (Pa.s)
*η_B_*	biaxial extension viscosity (Pa.s)
*η_E_*	uniaxial extension viscosity (Pa.s)
*η_P_*	planar extension viscosity (Pa.s)
*η*′*_L_*	large strain rate viscosity (Pa.s) (from the LAOS tests)
*η*′*_M_*	minimum strain rate viscosity (Pa.s) (from the LAOS tests)
*N_T_*	Trouton number (-)
*P/L*	Alveograph peak height to curve length ratio (-)
π	Pi number (=3.14)
r	radius of a cylinder (mm) (for the cylinder geometry in rheo-baking tests)
r	relaxation exponent (0 < r < 1, from stress relaxation tests)
*r_2_*	retardation time (s) (from non-linear creep recovery tests)
*RH*	relative humidity (%)
S	stiffness of the matter (-) (from stress relaxation tests)
*S*	strain stiffening ratio (-) (from the LAOS tests)
SAOS	Small Amplitude Oscillatory Shear
*SHI*	strain hardening index (from biaxial extension tests)
*σ*(*t*)	shear stress (Pa)
*T*	shear thickening ratio (-) (from the LAOS tests)
T	temperature (°C)
*t*	time (s, min)
*tanδ*	tangent phase angle (-)
*V*	volume of a cylinder (mm^3^) (for the cylinder geometry in rheo-baking tests)
*v*_3_/*v*_1_	the ratio of the third-order viscous Chebyshev coefficient to the first-order viscous Chebyshev coefficient (-) (from the LAOS tests)
*W*	work of deformation until rupture (J) (from Alveograph tests)
*ω*	frequency (rad/s, Hz)
